# Utilization and Costs of Gender-Affirming Care in a Commercially Insured Transgender Population

**DOI:** 10.1017/jme.2022.87

**Published:** 2022

**Authors:** Kellan Baker, Arjee Restar

**Affiliations:** 1: WHITMAN-WALKER INSTITUTE, WASHINGTON, DC, USA; 2: JOHNS HOPKINS BLOOMBERG SCHOOL OF PUBLIC HEALTH, BALTIMORE, MD, USA; 3: CENTER FOR APPLIED TRANSGENDER STUDIES, CHICAGO, IL, USA; 4: UNIVERSITY OF WASHINGTON, SEATTLE, WA, USA.

**Keywords:** Transgender, Cost, Utilization, Gender Affirmation, Health Equity

## Abstract

Many transgender people need specific medical services to affirm their gender. Gender-affirming health care services may include mental health support, hormone therapy, and reconstructive surgeries. Scant information is available about the utilization or costs of these services among transgender people, which hinders the ability of insurance regulators, health plans, and other health care organizations to plan and budget for the health care needs of this population and to ensure that transgender people can access medically necessary gender-affirming care. This study used almost three decades of commercial insurance claims from a proprietary database containing data on more than 200 million people to identify temporal trends in the provision of gender-affirming hormone therapy and surgeries and to quantify the costs of these services.

## Introduction

Transgender people have a gender identity that is different from the sex they were assigned at birth, and many seek multiple ways to access and attain gender affirmation across their lifetime.^1^ Gender affirmation refers to the multifaceted ways in which one may attain recognition of their gender socially (by publicly expressing their gender), psychologically (by rejecting internalized transphobia), legally (by correcting their gender marker and name on identification documents and records), and medically (by pursuing medical interventions like hormones or surgery).^2^ Gender affirmation is a non-linear, non-prescriptive pathway that is tailored to individual goals and affirmation needs, and it has been linked to multiple positive health outcomes such as better quality of life;^3^ lower rates of mental health conditions such as depression, anxiety, and psychological distress;^4^ decrease in or elimination of distress associated with gender dysphoria; and mitigation of stigma.^5^

In the context of medical interventions, the Standards of Care for Transgender and Gender Diverse People maintained by the World Professional Association for Transgender Health (WPATH) have established categories of health services and procedures that are recognized as gender-affirming medical care. These services include psychological support, hormone therapy, and reconstructive surgeries.^6^ Hormone therapy typically involves estrogens and anti-androgens for transgender women and other transfeminine people and testosterone for transgender men and other transmasculine people. Surgeries that may be part of gender affirmation for transgender people include genital surgeries, such as phalloplasty or vaginoplasty; gonadectomy; chest surgeries, including mastectomy or mammoplasty; and facial surgeries, particularly for transgender women.

There are multiple structural and economic barriers that transgender people face when seeking gender-affirming medical services and procedures. Compared to the general US population, transgender people are more likely to be uninsured (14% vs. 11%), unemployed (15% vs. 5%), and living in poverty (29% vs. 12%).^7^ Even for people with insurance, reports of insurance denials are common,^8^ and many people report that deductibles and other out-of-pocket costs like copays and coinsurance for hormones and surgeries are a major economic barrier to pursuing gender-affirmation care.^9^ One study using Centers for Medicare and Medicaid Services prescription drug plan formulary files found that out-of-pocket costs for gender-affirming hormone therapy can be substantial, ranging between $84 to $2,716 in 2010 and from $72 to $3,792 in 2018.^10^ Moreover, insurers often require proof of referral letters for hormone initiation as well as surgical procedures from mental health professionals, which can also serve as a limiting factor given the inadequate workforce capacity of gender-affirming therapists, counselors, social workers, primary care providers, and surgeons, particularly in geographical areas that are prone to insurance network inadequacy issues and policy restrictions in the US.^11^

The objective of the present study was to investigate temporal trends in coding, utilization, and costs of gender-affirming hormone therapy and surgeries using a proprietary commercial insurance claims database that captures all encounters for enrolled beneficiaries.

As a step to providing coverage of gender-affirming care, one imperfect approach has been to characterize a need for gender-affirming care using diagnoses such as gender dysphoria, which replaced gender identity disorder in the fifth edition of the Diagnostic and Statistical Manual of Mental Disorders (DSM-5).^12^ This change, like the revision to the *International Classification of Diseases*, 11th Revision (ICD-11) to create a new diagnosis of gender incongruence (codes: HA60, HA61, HA6Z), clarifies that the target of gender-affirming medical interventions is not the person’s gender identity itself but rather the clinically significant distress that can accompany a lack of alignment between gender identity and sex assigned at birth.^13^

Over the last decade, interest among insurance carriers, regulators, and medical coders about trends in gender-affirming care has grown as nondiscrimination laws and private employer practices have evolved toward ensuring coverage for and broadening the availability of these services.^14^ Because no national health survey consistently asks questions about gender identity, efforts to track trends and measure the effects of coverage changes have focused on alternative sources of data, such as insurance claims.^15^ The objective of the present study was to investigate temporal trends in coding, utilization, and costs of gender-affirming hormone therapy and surgeries using a proprietary commercial insurance claims database that captures all encounters for enrolled beneficiaries. We anticipated that transgender people in this database would be identified in all geographic regions and that claims for hormone therapy and gender-affirming surgeries would come from diverse clinical specialties and would increase over time, particularly in the period after 2010, when the Affordable Care Act (ACA) made private health insurance broadly more accessible and both public and private payers began to remove coverage exclusions of gender-affirming care.^16^ We also expected that the age at which transgender people were first identified in the database would drop over time in parallel with general U.S. population trends, which have shown increasing numbers of people identifying as transgender at younger ages.^17^ Finally, we anticipated that the system-wide costs of gender-affirming care would increase over time as insurance coverage of these services became more common, but that the impact of covering gender-affirming care on payers’ budgets would be small.

## Methods

### Data

We accessed the OptumLabs Data Warehouse (OLDW), which contains insurance claims data for more than 200 million people covered by commercial and Medicare Advantage plans. The OLDW Unified View provides nationwide de-identified physician, facility, and pharmacy claims, as well as person-level enrollment and demographic information. Claims include ICD-9 and ICD-10 diagnostic codes (up to five codes for physician claims and up to nine codes plus any admitting diagnosis, if present, for facility claims), Healthcare Common Procedure Coding System (HCPCS) and Current Procedural Terminology (CPT) codes, health plan and patient paid amounts, type of facility, provider type, and an internal provider identification number. OLDW is a closed system that captures complete records of health service utilization during periods of enrollment. Claims data are refreshed monthly and are accessible for research after a six-month lag. The demographic information in the Unified View is year of birth, recorded sex, census region, and race/ethnicity. Race/ethnicity is imputed through a proprietary process by a third party and provided to OLDW for use in analyses. Most fields are 100 percent populated, with the exception of imputed race/ethnicity, which is approximately 70 percent complete. Individuals receive a unique identifier and can be followed over time whenever they are enrolled in coverage.

### Study Population

Using an approach developed by researchers at the Veterans Administration (VA) and elsewhere,^18^ we identified transgender people by searching OLDW for transgender-specific diagnostic codes in all physician and facility claims of people with simultaneous commercial medical and pharmacy coverage. Medicare Advantage enrollees were not included. Before the U.S. conversion to ICD-10 in mid-2015, we searched for the following ICD-9 codes in any diagnosis position: transsexualism (302.5x), gender identity disorder in children (302.6), and gender identity disorder in adolescents and adults (302.85). In 2015 and later, we added the following ICD-10 codes in any position: transsexualism (F64.0); gender identity disorder in adolescence or adulthood (F64.1); gender identity disorder in childhood (F64.2); other gender identity disorders (F64.8); gender identity disorder, unspecified (F64.9); and personal history of sex reassignment (Z87.890).^19^ To improve specificity, we required two instances of at least one code separated by 30 days in the claims history.^20^ The first appearance of any transgender-specific diagnosis code in a person’s claims history was designated as their index date of diagnosis, which was used to assess trends in the age at which people received their first transgender-specific code in the OLDW database. Research indicates that a child’s sense of gender identity typically develops around the age of three, so we excluded children who were younger than three on their index date.^21^

### Covariates

To assess trends in prescribing patterns, we extracted the transgender-specific diagnostic codes assigned on each person’s index date, along with the demographic variables of year of birth, race/ethnicity, region, and recorded sex. We categorized age in 2021 as 4-17, 18-29, 30-39, 40-49, 50-59, 60-69, and 70+; race/ethnicity as white, Black, Asian, Hispanic, or unknown; and location by census region (Northeast, South, Midwest, West). Recorded sex was either male or female; OLDW contains very few instances of sex being recorded as “unknown,” so we dropped those rare cases. It was impossible to know whether this variable referred to gender identity or to sex assigned at birth, so while it was included as a covariate, it should not be interpreted as a true estimate of the proportions of transmasculine and transfeminine people in the database. To assess patterns in use of transgender-specific diagnostic codes by specialty, we also extracted the internal OLDW identification number and specialty of clinicians who assigned these codes in any encounter, regardless of whether it was the index diagnosis.

### Outcome Measures

Following published guidelines for hormone therapy in transgender people,^22^ we characterized gender-affirming testosterone therapy as at least one pharmacy claim for any formulation of testosterone without any claim for an estrogen formulation; for transgender women and other transfeminine people, gender-affirming hormone therapy was at least one claim for an estrogen formulation with at least one claim for an anti-androgen such as spironolactone or bicalutamide. Dutasteride and finasteride, which may be used by transfeminine people for purposes of gender affirmation but also by transmasculine people to prevent hair loss associated with testosterone use, were not included.^23^ We classified people with claims for both testosterone and estrogen formulations as transmasculine because of the potential use of estrogen formulations for birth control among people assigned female at birth, regardless of gender identity. We did not use recorded sex data to classify hormone therapy because it was impossible to determine whether the sex variable in the database referred to current gender identity or to assigned sex at birth. Gonadotropin-releasing hormone (GnRH) analogs, which may be prescribed to transgender adolescents of any gender to delay the onset of puberty as a precursor to eventual hormone replacement therapy with testosterone or estrogens, were included as a separate category. For each gender-affirming hormone therapy claim, we extracted the generic and brand names, dosage, out-of-pocket and health plan paid amounts, and the prescribing provider’s specialty and internal OLDW identification number (Appendix A, Table A.1).

To identify gender-affirming surgeries, we first extracted all physician and facility claims that included a transgender-specific ICD-9 or ICD-10 diagnostic code in any position. We then used published coverage protocols^24^ to identify claims with ICD-9 or ICD-10 procedure codes or CPT codes that can be used to bill for the following categories of gender-affirming surgical procedures: phalloplasty or metoidioplasty, hysterectomy, and mastectomy for transgender men and other transmasculine people and vaginoplasty, orchiectomy, mammoplasty, and facial feminization for transgender women and other transfeminine people (Appendix A, Table A.2). Codes that could not be readily associated with a specific gender were grouped as “unspecified top surgery” (i.e., mastectomy or mammoplasty) or “unspecified genital surgery” (i.e., phalloplasty/metoidioplasty or vaginoplasty). We confirmed the composition of this code list with a surgeon who performs high volumes of these procedures (Loren Schecter, personal communication, August 20, 2019).

### Descriptive Analyses

We calculated the incidence by year of transgender people newly identified in OLDW using their index date. The denominator for both annual incidence and the total number of transgender people with coverage by year was the count of all people with commercial coverage in OLDW in that year. We explored trends in coding by calculating the mean age at index diagnosis for people with index dates between 1993 to 2000, 2001 to 2010, and 2011 to 2020, as well as by assessing the relative proportions of transgender-specific diagnostic codes assigned by each clinical specialty. We used χ^2^ tests to compare index codes by demographics.

The assessment of gender-affirming health services utilization consisted of annual counts of individual hormone therapy prescriptions in each category (testosterone, estrogens plus anti-androgens, and GnRH analogs), annual counts of the number of people receiving any gender-affirming hormone therapy prescription, counts of episodes of individual surgical procedures in each category of surgeries by year, and annual counts of transgender people who underwent any gender-affirming surgical procedure. Procedures that occurred within 14 days of each other were counted as a single episode. We calculated the percentage of people who received hormone therapy or a surgical procedure among all individuals identified as transgender in the database who were enrolled in coverage for any part of each year. We used multivariable logistic regression models to identify demographic characteristics associated with receipt of hormone therapy or gender-affirming surgery. Statistical significance was set at α = 0.05, and analyses were conducted in R (version 4.0.2).

Annual costs for each category of hormone therapy were calculated from a payer perspective by summing the health plan paid costs; we also calculated average annual health plan paid costs per person for each category. Average and annual costs for each type of surgery were similarly calculated from a payer perspective, and all costs incurred during the 14-day window after each procedure were included. We calculated the annual budget impact of the overall cost of gender-affirming care, including all types of hormone therapy and surgical procedures, using the total OLDW population with commercial coverage in each year as the denominator. All costs were estimated in 2019 dollars.

## Results

**Figure 2.1:**
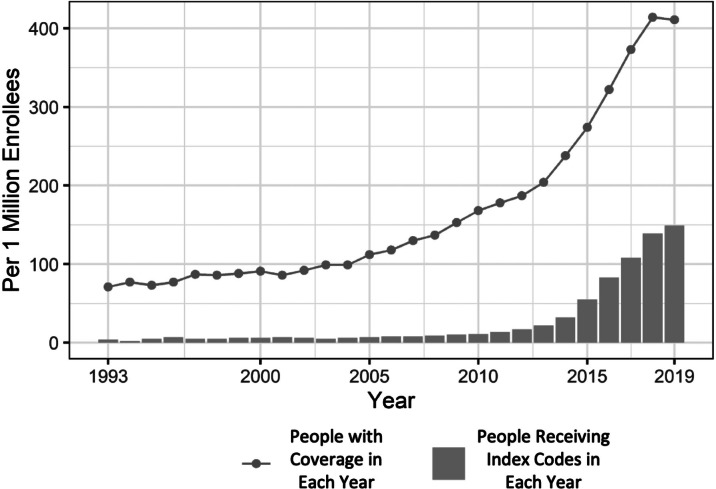
Annual New Identifications and Total Count of Transgender People in the OptumLabs Data Warehouse, 1993-2019

**Figure 2.2:**
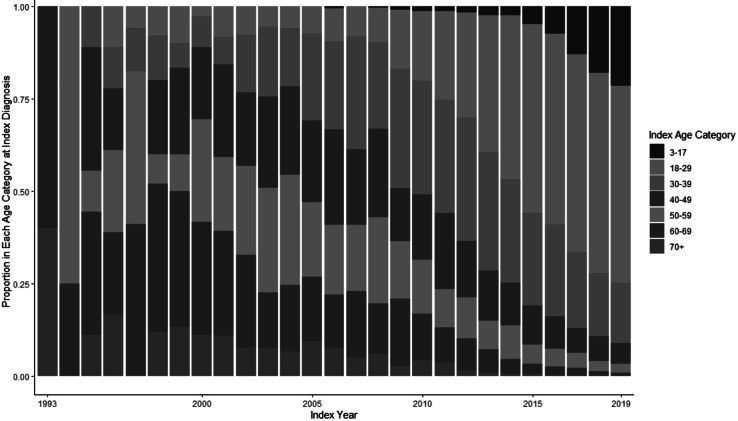
Age Distribution of Newly Identified Transgender People by Index Year, 1993-2019

We identified 16,619 people who had physician or facility claims and met our inclusion criteria between 1993 and 2019. Of this group, 15,790 also had pharmacy claims. The annual incidence of index codes, meaning the appearance of an individual’s first transgender-specific code in the database, rose from 4 per million enrollees in 1993 to 149 per million in 2019, with more than 80 percent of that growth occurring between 2011 and 2019. Between 1993 and 2000 and between 2001 and 2010, an average of 18 and 166 people, respectively, received a transgender-specific code for the first time each year; between 2011 and 2019, an average of 1,646 people were newly identified as transgender in OLDW each year. The number of people in OLDW with transgender codes in each year similarly increased slowly through the first two decades before beginning an exponential rise around 2011 (Figure 2.1). In 1993, the number of transgender people with coverage in OLDW was 71 per million enrollees; this number rose slowly to 178 per million in 2010 before climbing rapidly to 411 per million by 2019. The mean age at index diagnosis declined from 33.9 years in 1993 to 26.3 years in 2019 (Figure 2.2). The transgender population was young, with the largest proportion (46%) in the age group between 18 and 29 as of 2019. The majority were identified in the database as female (53%) and white (67%), and most (35%) lived in the South, where OLDW has large representation (Table 2.1).

**Table 2.1:** Demographics of Transgender People Identified in the OptumLabs Data Warehouse, 1993-2019

Characteristic	Transgender Population (N = 16,619)
*Age, years*	
Mean (SD)	30.6 (13.4)
Range	4 – 87
*Age group in years, % (n)*	
4-17	12 (1,961)
18-29	46 (7,703)
30-39	21 (3,525)
40-49	9.5 (1,578)
50-59	6.1 (1,013)
60-69	4.0 (671)
70+	1.0 (168)
*Age at index diagnosis, years*	
Overall mean (SD)	27.2 (11.8)
Overall range	3 – 79
Mean, 1993-2000	33.9
Mean, 2001-2010	34.5
Mean, 2011-2019	26.3
Recorded Sex, % (n)	
Female	53 (8,864)
Male	47 (7,755)
*Imputed Race/Ethnicity, % (n)*	
Asian	2.7 (452)
Black	8.1 (1,339)
Hispanic	8.0 (1,322)
White	67 (11,123)
Unknown	14 (2,383)
Region, % (n)	
Midwest	28 (4,675)
Northeast	11 (1,820)
South	35 (5,826)
West	26 (4,298)
*Days of Coverage*	
Mean (SD)	1,664 (1,453)
Range	30 – 9,861

**Figure A.1:**
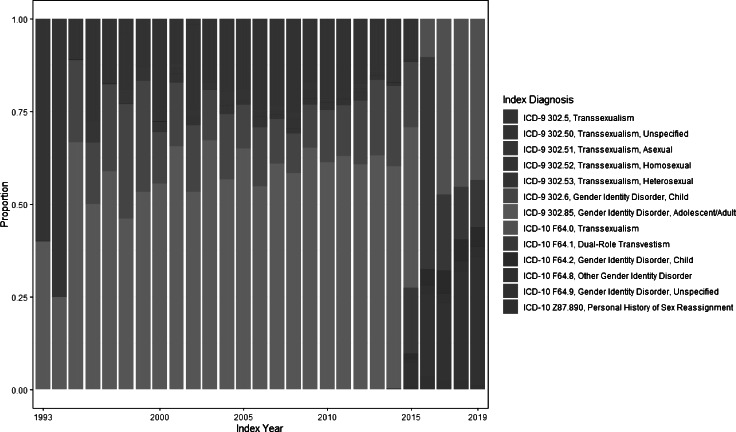
Index Codes by Year, 1993-2019

**Figure A.2:**
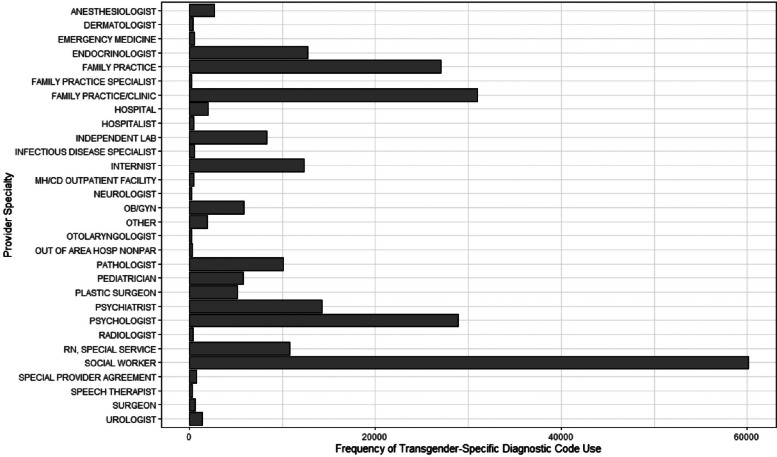
Use of Transgender-Specific Codes by Provider Specialty

The most common index code during the ICD-9 period was the non-specific code 302.85 (Gender Identity Disorder in Adolescents or Adults). Codes with sexual orientation subclassification (e.g., 302.53; Transsexualism, Heterosexual Sexual History) became less common throughout the ICD-9 period; these codes were phased out in the conversion to ICD-10 in mid-2015 (Appendix A, Figure A.1). Immediately following the conversion, there was a temporary spike in the use of F64.1 (Dual-Role Transvestism). There was no increase over time in the use of codes specific to children (e.g., F64.2, Gender Identity Disorder in Childhood). While transgender-specific diagnostic codes typically appeared in claims for services that could be part of gender affirmation, including mental health counseling as well as hormone therapy and surgeries, the use of these codes was not confined to gender-affirming care: these codes were also identified in claims for encounters such as arthroscopic knee surgeries and influenza vaccines. The provider specialties that used these codes most often were social work, family practice, and psychology (Appendix A, Figure A.2).

**Figure 2.3:**
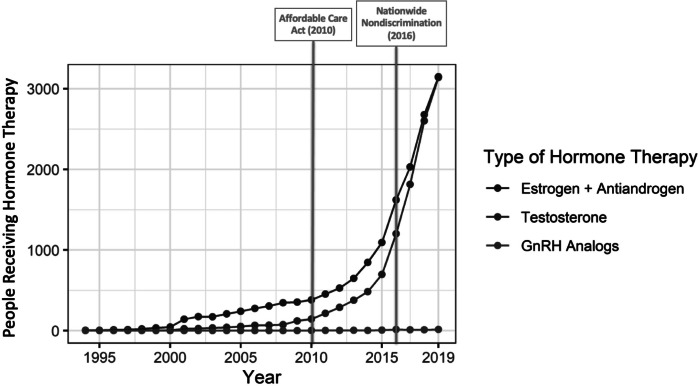
Number of People with Claims for Gender-Affirming Hormone Therapy by Year and Medication Type, 1993-2019

**Figure 2.4:**
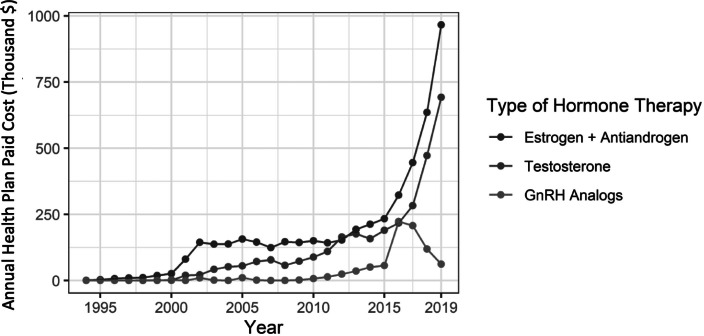
Annual Health Plan Paid Cost by Gender-Affirming Hormone Therapy Type, 1993-2019

Seventy-two percent of transgender people had at least one encounter for gender-affirming hormone therapy. The clinical specialties most likely to write prescriptions for hormone therapy were family practice (28%) and endocrinology (18%) (Appendix A, Table A.3). Many individual providers were represented, and no single provider wrote more than 1.6 percent of all the prescriptions in the claims database. Hormone therapy by gender was roughly even between transmasculine and transfeminine regimens: 46 and 54 percent of people on hormone therapy were classified as transmasculine or transfeminine, respectively. Only 0.4 percent of those on hormone therapy were observed to have received GnRH treatment, and 78 percent of those who had been on GnRH treatment subsequently received prescriptions for estrogens or testosterone. While the number of people on GnRH treatment remained consistently low, the number of people receiving hormone therapy with estrogen or testosterone increased rapidly beginning around 2011 (Figures 2.3 and 2.4). In 2011, 17 percent of transgender people identified in this database were receiving gender-affirming hormone therapy, and by 2019 this proportion had increased almost 4-fold, to 65 percent. The average payer costs of gender-affirming hormones were consistently low for both testosterone and estrogen therapy, at $121 and $153 per year; GnRH therapy cost an average of $2,410 per person per year (Table 2.2). As a proportion of total costs, out-of-pocket spending per year was 38 percent for estrogens, 25 percent for testosterone, and 8 percent for GnRH.

**Table 2.2:** Frequency and Costs of Claims for Gender-Affirming Hormone Therapy, 1993-2019

	Testosterone	Estrogens + Anti-Androgens	GnRH	All Combined
Overall frequency	5,522	6,403	51	11,976
Percent of hormone therapy users	46.1	53.5	0.43	-
Percent of total transgender population (N = 16,619)	33.2	38.5	0.31	72.1
Average time in coverage (years)	4.5	4.8	6.8	-
*Health plan paid costs, $*				
Per person	545	735	16,385	515*
Per year of coverage	121	153	2,410	107*
Out-of-pocket costs, $				
Per person	178	458	1,453	240*
Per year of coverage	40	95	214	51*
*Total costs, $*				
Per person	723	1,193	17,838	755*
Per year of coverage	161	248	2,623	157*
Out-of-pocket proportion, %	25	38	8	32

GnRH = gonadotropin-releasing hormone

* Average weighted by proportion of people with prescriptions for each type of therapy; denominator is the total number of transgender people identified in OLDW (N = 16,619)

**Figure 2.5:**
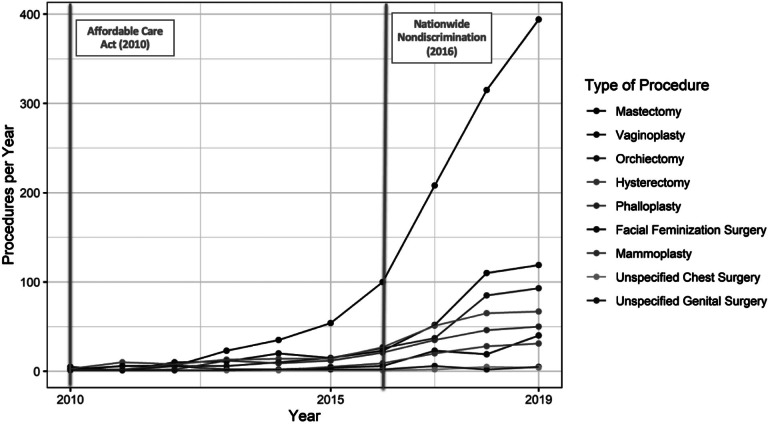
Number of Gender-Affirming Surgical Procedures by Year, 2010-2019

**Figure 2.6:**
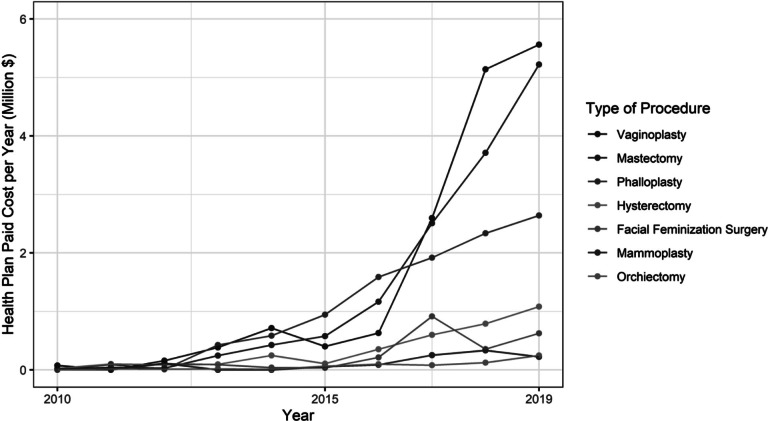
Annual Health Plan Paid Cost by Gender-Affirming Procedure Type, 2010-2019

Temporal trends in the frequency of gender-affirming surgeries paralleled those of hormone therapy. Throughout the first two decades of claims, gender-affirming surgeries were performed infrequently, if at all, but the annual number of procedures performed began to increase around 2011: in 2011, 21 people (0.5% of all transgender people with coverage that year) underwent a gender-affirming surgery, and by 2019, that number had risen to 794 (8%) (Figures 2.5 and 2.6). Overall, 14 percent of the transgender people identified for this analysis had ever undergone a gender-affirming surgery while enrolled in OLDW, of which mastectomy was the most common procedure. The per-episode payer costs of gender-affirming surgeries ranged from $6,927 for orchiectomy to $45,080 for vaginoplasty and $63,432 for phalloplasty (Table 2.3). As vaginoplasty and phalloplasty were frequently multi-episode procedures, the total average cost of these procedures per person was $53,645 and $133,911, respectively. There were substantially lower odds of having undergone surgery among people living in the South (adjusted odds ratio [OR]: 0.74, 95% confidence interval [CI]: 0.63, 0.88), although there were no differences by imputed race (Appendix A, Table A.4). No single provider was responsible for more than 6.6 percent of surgeries.

**Table 2.3:** Frequency and Costs of Claims for Gender-Affirming Surgeries, 1993-2019

	Phalloplasty	Vaginoplasty	Hysterectomy	Orchiectomy	Mastectomy	Mammoplasty	FFS	Total
Total procedures (n)	195	392	311	289	1,163	113	105	2,568
Total people (n)	94	326	309	288	1,128	105	84	2,334*
People as percent of total transgender population (N = 16,619)	0.6	2.0	1.9	1.7	6.8	0.6	0.5	14.0
*Mean health plan paid costs, $*								
Per procedure	63,432	45,080	14,433	6,927	12,304	16,164	28,934	26,753
Per person	133,911	53,645	14,538	6,927	12,680	17,426	35,316	39,206
*Mean out-of-pocket costs, $*								
Per procedure	1,886	2,205	1,617	1,250	2,177	1,134	1,029	1,614
Per person	3,982	2,624	1,629	1,250	2,244	1,223	1,256	2,030
*Mean total costs, $*								
Per procedure	65,318	47,285	16,050	8,177	14,481	17,298	29,963	28,367
Per person	137,893	56,269	16,167	8,177	14,924	18,649	36,572	41,236
Out-of-pocket proportion per person, %	2.9	4.7	10.1	15.3	15.0	6.6	3.4	4.9

Over the time period covered by this study, the annual frequency of gender-affirming hormone therapy and surgeries increased both in absolute terms and as a proportion of the number of transgender people identified in the database, and costs changed accordingly. In 2019, each covered transgender person incurred an average of $1,776 in costs for gender-affirming hormone therapy and surgeries combined. Considered on a per-member basis across the entire commercially insured population in OLDW, the budget impact of gender-affirming care in 2019 was $0.73 per year, or $0.06 per member per month (PMPM).

## Discussion

To our knowledge, this is the first study to evaluate temporal trends in coding, utilization, and costs for both gender-affirming hormone therapy and surgeries. We found that the number of people receiving transgender-specific diagnostic codes and accessing gender-affirming care in this privately insured population has increased rapidly over the decade between 2011 and 2019. Even as coverage of gender-affirming care has expanded, its budget impact remains small: the PMPM estimate of providing gender-affirming care in 2019 was $0.06 when distributed across all people with commercial coverage in OLDW. This is in line with estimates from a cost-effectiveness study that estimated the costs of coverage for gender-affirming care at $0.016 when spread across the entire U.S. population.^25^

These trends in utilization of gender-affirming health services align with broader societal trends in the visibility of transgender people. The time frame of this increase coincides with policy reforms over the last decade lifting several barriers that previously limited both the use of transgender-specific codes and the provision of gender-affirming care. In 2010, the ACA introduced new guaranteed-issue protections in private insurance that were interpreted by the U.S. Department of Health and Human Services (HHS) to prohibit the designation of a transgender identity as a pre-existing condition for which insurance coverage could be restricted or denied.^34^ Between 2010 and 2014, HHS promulgated several regulations that codified nondiscrimination protections on the basis of gender identity in insurance marketing, benefit design, and coverage determinations.^26^ Around the same time, individual states began to adopt or strengthen similar protections by interpreting existing law to prohibit unfair discrimination against transgender people in both state-regulated health insurance markets and state Medicaid programs.^27^ These reforms included the 2014 rescission of Medicare’s ban on coverage for gender-affirming surgeries and a 2016 HHS regulation that prohibited blanket exclusions of gender-affirming care in both public and private coverage.^28^ Though the Trump administration revised that regulation in 2020 and future activity by the Biden administration remains unknown, state and federal courts have consistently found that discrimination against transgender people on the basis of gender identity is a form of sex discrimination.^29^ As of early 2021, 24 states and territories prohibited blanket transgender coverage exclusions in state-regulated private coverage, up from one in the pre-2010 period.^30^ The biggest increase in the number of people being identified as transgender in OLDW in the decade between 2010 and 2020 occurred in the South, where no states apart from Virginia, Maryland, and Delaware have state-specific protections. This pattern is consistent with the hypothesis that the 2016 national regulation played a substantial role in removing barriers to private coverage for transgender people, though more research is needed to explore this possibility.

The findings of this study indicate that the impact of gender-affirming care on payer budgets has remained nominal even as national trends in coverage policies have made this care more accessible to transgender people. Future directions for research include assessing the health outcomes associated with access to gender-affirming care, improving methods for identifying transgender people in insurance claims databases, and investigating opportunities to link different data sources to provide a more complete picture of the health needs and experiences of transgender people.

As restrictions on coverage for gender-affirming care have receded, other studies using data sources such as the National Inpatient Sample have identified increases in the number of gender-affirming surgeries performed in the U.S.^31^ The present study expands this evidence base by analyzing the frequency of individual procedures and assessing trends in hormone therapy use as well; a better understanding of the availability and uptake of both gender-affirming surgeries and hormone therapy is important for insurance carriers seeking to ensure the adequacy of their coverage and provider networks for these services and for hospitals and other health service organizations identifying trends in patient care needs. These data may also help federal and state insurance regulators establish baseline estimates of service availability and utilization, which can be used to monitor market conduct and identify potential concerns related to inadequacy of benefit designs or inappropriate use of utilization management tools. For instance, this study found that utilization of GnRH treatment remained low, even as the number of people identified in the 4-17 age group increased. This pattern is consistent with reports that barriers in insurance coverage of GnRH treatment for transgender adolescents remain high.^32^ Some regulators are beginning to explore the degree to which restrictions on coverage of GnRH treatment for this population may violate nondiscrimination requirements on the basis of gender identity and age.^33^

## Limitations

This study has several limitations, many of which relate to the difficulty of using diagnoses in insurance claims as proxies for gender identity, which is a complex aspect of personal identity that has social, legal, and medical components. Because this insurance claims database does not currently include any self-reported data on gender identity, it was not possible to determine how many people in the database would self-identify as transgender but are not captured by the algorithm based on transgender-specific diagnostic codes. The proportion of the population in this database that was identified as transgender was 411 per million in 2019 (0.04%), which is comparable to other estimates from clinical records but much less than estimates from more representative population surveys that use self-report, which range between 0.1 percent and 2.0 percent.^34^ It was also impossible to definitively identify claims for gender-affirming care, as the assessment of coding practices indicated that these codes may be applied to services provided to transgender people that do not have any relation to gender affirmation. We thus may have incorrectly categorized unrelated services as gender-affirming care; this was a particular concern with services that may be more commonly needed for other indications, such as hysterectomy and estrogen therapy.

At the same time, we may have missed services and procedures that were provided for purposes of gender affirmation but were not submitted with transgender-specific diagnostic codes. The number of claims with procedure codes that might indicate a gender-affirming service but that were not coded with relevant diagnostic codes was very small among the group of people identified as transgender, but it was not possible to know how many such procedures for purposes of gender affirmation were performed for people who were not included in the transgender group. The routine capture of self-reported gender identity data in clinical records, including both medical records and claims, would aid in assessments of transgender population size and health services costs and use. Similarly, more consistent coding standards guiding the application of both diagnostic codes related to a need for gender-affirming care and procedure codes describing the provision of this care would improve estimates of the frequency and costs of these procedures.

## Conclusion

The number of people with transgender-specific diagnostic codes in this commercial insurance claims database has increased sharply over the last decade, in tandem with law and policy changes that seek to remove barriers to coverage for this population. In 2019, almost 10,000 people were identified as transgender in this database, representing 0.04 percent of people with commercial coverage in OLDW. In the same year, 65 percent of people identified as transgender were receiving gender-affirming hormone therapy, and 8 percent had some gender-affirming surgical procedure. The annual cost of providing gender-affirming care for this population was $1,776 per person, or $0.06 per member per month. The findings of this study indicate that the impact of gender-affirming care on payer budgets has remained nominal even as national trends in coverage policies have made this care more accessible to transgender people. Future directions for research include assessing the health outcomes associated with access to gender-affirming care, improving methods for identifying transgender people in insurance claims databases, and investigating opportunities to link different data sources to provide a more complete picture of the health needs and experiences of transgender people.

## Note

The authors have no conflicts to disclose.

## Supplementary material

For supplementary material accompanying this paper visit https://doi.org/10.1017/jme.2022.87. click here to view supplementary material

## **Appendix A:** Supplementary Material

**Table A.3:** Hormone Therapy Prescriptions by Provider Specialty

Specialty Category	Specialty Total	Proportion of Total, %
Family Practice	59,536	27.7
Endocrinology	37,702	17.5
Internal Medicine	30,753	14.3
Registered Nurse	28,794	13.4
Obstetrics and Gynecology	16,964	7.9
Other	11,510	5.4
Pediatrics	6,499	3.0
Infectious Disease	1,491	0.7
Psychiatry	1,433	0.7

**Table A.4:** Associations Between Demographic Characteristics and Gender-Affirming Medical Services

	HORMONE THERAPY			SURGERIES		
Characteristic	OR	95% CI	p-value	OR	95% CI	p-value
*Imputed Race*						
White	Ref	--	--	--	--	--
Asian	1.13	0.90, 1.42	0.3	1.19	0.89, 1.56	0.2
Black	1.21	1.05, 1.39	0.009	1.09	0.91, 1.29	0.3
Hispanic	0.93	0.81, 1.06	0.3	0.96	0.80, 1.15	0.7
Unknown	0.93	0.84, 1.03	0.2	0.80	0.69, 0.93	0.004
*Geography*						
Northeast	Ref	--	--	--	--	--
Midwest	0.93	0.82, 1.05	0.3	0.97	0.82, 1.15	0.7
South	1.20	1.06, 1.35	0.004	0.74	0.63, 0.88	<0.001
West	1.28	1.12, 1.45	<0.001	0.96	0.81, 1.14	0.6
*Age Group*						
4-17	0.17	0.15, 0.18	<0.001	0.02	0.01, 0.04	<0.001
18-29	Ref	--	--	--	--	--
30-39	1.43	1.30, 1.59	<0.001	1.43	1.28, 1.60	<0.001
40-49	1.27	1.12, 1.45	<0.001	1.24	1.06, 1.45	0.007
50-59	1.10	0.95, 1.29	0.2	1.11	0.91, 1.35	0.3
60-69	1.20	0.99, 1.45	0.064	0.59	0.43, 0.78	<0.001
70+	1.05	0.75, 1.52	0.8	0.43	0.20, 0.79	0.013
*Recorded Sex*						
Female	Ref	--	--	--	--	--
Male	0.88	0.82, 0.95	<0.001	0.77	0.70, 0.85	<0.001

OR = odds ratio, CI = confidence interval, Ref = reference category
