# Supplementary material for: Utilization and Costs of Gender-Affirming Care in a Commercially Insured Transgender Population
Source: J Law Med Ethics. 2022 Fall;50(3):456–70. doi: 10.1017/jme.2022.87 (PMC9679590; doi:10.1017/jme.2022.87)
Supplement: Supplementary file 1 [file S1073110522000870sup001.pdf]

Appendix A: Supplementary Tables

Table A.1
Codes for Gender-Affirming Hormone Therapy

GENERIC NAME	BRAND NAME
**Testosterone Formulations**	
METHYLTESTOSTERONE	ANDROID
METHYLTESTOSTERONE	ANDROID-10
METHYLTESTOSTERONE	METHITEST
METHYLTESTOSTERONE	METHYLTESTOSTERONE
METHYLTESTOSTERONE	TESTRED
TESTOSTERONE	ANDRODERM
TESTOSTERONE	ANDROGEL
TESTOSTERONE	AXIRON
TESTOSTERONE	FORTESTA
TESTOSTERONE	NATESTO
TESTOSTERONE	STRIANT
TESTOSTERONE	TESTIM
TESTOSTERONE	TESTODERM
TESTOSTERONE	TESTODERM TTS
TESTOSTERONE	TESTOPEL
TESTOSTERONE	TESTOSTERONE
TESTOSTERONE CYPIONATE	DEPO-TESTOSTERONE
TESTOSTERONE CYPIONATE	TESTOSTERONE CYPIONATE
TESTOSTERONE ENANTHATE	DELATESTRYL
TESTOSTERONE ENANTHATE	TESTOSTERONE ENANTHATE
TESTOSTERONE ENANTHATE	XYOSTED
TESTOSTERONE MICRONIZED	TESTOSTERONE MICRONIZED
TESTOSTERONE PROPIONATE	FIRST-TESTOSTERONE MC
TESTOSTERONE PROPIONATE	TESTOSTERONE PROPIONATE
**Estrogen or Anti-Androgen Formulations**	
BICALUTAMIDE	CASODEX
BICALUTAMIDE	BICALUTAMIDE

The Journal of Law, Medicine & Ethics, 50 (2022): A1-A44. © 2022 The Author(s)

Table A.1 (continued)
Codes for Gender-Affirming Hormone Therapy

GENERIC NAME	BRAND NAME
DESOG-E.ESTRADIOL/E.ESTRADIOL	AZURETTE
DESOG-E.ESTRADIOL/E.ESTRADIOL	VIORELE
DESOG-E.ESTRADIOL/E.ESTRADIOL	BEKYREE
DESOG-E.ESTRADIOL/E.ESTRADIOL	DESOGESTR-ETH ESTRAD ETH ESTRA
DESOG-E.ESTRADIOL/E.ESTRADIOL	KARIVA
DESOG-ET ESTRA/ETHIN ESTRA	VIORELE
DESOG-ET ESTRA/ETHIN ESTRA	KARIVA
DESOG-ET ESTRA/ETHIN ESTRA	AZURETTE
DESOGESTREL-ETHINYL ESTRADIOL	APRI
DESOGESTREL-ETHINYL ESTRADIOL	ISIBLOOM
DESOGESTREL-ETHINYL ESTRADIOL	DESOGEN
DESOGESTREL-ETHINYL ESTRADIOL	ORTHO-CEPT
DESOGESTREL-ETHINYL ESTRADIOL	EMOQUETTE
DESOGESTREL-ETHINYL ESTRADIOL	ENSKYCE
DESOGESTREL-ETHINYL ESTRADIOL	JULEBER
DESOGESTREL-ETHINYL ESTRADIOL	CYRED
DESOGESTREL-ETHINYL ESTRADIOL	DESOGESTREL-ETHINYL ESTRADIOL
DESOGESTREL-ETHINYL ESTRADIOL	RECLIPSEN
DROSPIR/ETH ESTRA/LEVOMEFOL CA	DROSPIRENONE-ETH ESTRA-LEVOMEF
DROSPIR/ETH ESTRA/LEVOMEFOL CA	BEYAZ
DROSPIR/ETH ESTRA/LEVOMEFOL CA	SAFYRAL
ESTRADIOL	ALORA
ESTRADIOL	ESTRASORB
ESTRADIOL	ESTRACE
ESTRADIOL	VIVELLE
ESTRADIOL	ESCLIM
ESTRADIOL	EVAMIST
ESTRADIOL	VAGIFEM
ESTRADIOL	ESTRADERM
ESTRADIOL	YUVAFEM
ESTRADIOL	GYNODIOL
ESTRADIOL	DOTTI
ESTRADIOL	DIVIGEL

The Journal of Law, Medicine & Ethics, 50 (2022): A1-A44. © 2022 The Author(s)

GENERIC NAME	BRAND NAME
ESTRADIOL	IMVEXXY
ESTRADIOL	ESTRADIOL TRANSDERMAL PATCH
ESTRADIOL	ELESTRIN
ESTRADIOL	MINIVELLE
ESTRADIOL	VIVELLE-DOT
ESTRADIOL	CLIMARA
ESTRADIOL	ESTRADIOL
ESTRADIOL	ESTROGEL
ESTRADIOL	ESTRING
ESTRADIOL ACETATE	FEMRING
ESTRADIOL CYPIONATE	ESTRADIOL CYPIONATE
ESTRADIOL CYPIONATE	DEPO-ESTRADIOL
ESTRADIOL MICRONIZED	ESTRADIOL MICRONIZED
ESTRADIOL MICRONIZED	ESTRADIOL
ESTRADIOL VALERATE	ESTRADIOL VALERATE
ESTRADIOL VALERATE	DELESTROGEN
ESTRADIOL VALERATE/DIENOGEST	NATAZIA
ESTRADIOL/DROSPIRENONE	ANGELIQ
ESTRADIOL/LEVONORGESTREL	CLIMARA PRO
ESTRADIOL/NORETH AC	MIMVEY
ESTRADIOL/NORETH AC	ESTRADIOL-NORETHINDRONE ACETAT
ESTRADIOL/NORETH AC	ACTIVELLA
ESTRADIOL/NORETH AC	COMBIPATCH
ESTRADIOL/NORETHINDRONE ACET	ESTRADIOL-NORETHINDRONE ACETATE
ESTRADIOL/NORETHINDRONE ACET	COMBIPATCH
ESTRONE	ESTRONE
ESTROPIPATE	ESTROPIPATE
ESTROPIPATE	ORTHO-EST
ETHINYL ESTRADIOL	ESTINYL
ETHINYL ESTRADIOL	ETHINYL ESTRADIOL
ETHINYL ESTRADIOL/DROSPIRENONE	OCELLA
ETHINYL ESTRADIOL/DROSPIRENONE	LORYNA
ETHINYL ESTRADIOL/DROSPIRENONE	YASMIN 28

Table A.1 (continued)
Codes for Gender-Affirming Hormone Therapy

GENERIC NAME	BRAND NAME
ETHINYL ESTRADIOL/DROSPIRENONE	NIKKI
ETHINYL ESTRADIOL/DROSPIRENONE	SYEDA
ETHINYL ESTRADIOL/DROSPIRENONE	LO-ZUMANDIMINE
ETHINYL ESTRADIOL/DROSPIRENONE	VESTURA
ETHINYL ESTRADIOL/DROSPIRENONE	ZARAH
ETHINYL ESTRADIOL/DROSPIRENONE	YAZ
ETHINYL ESTRADIOL/DROSPIRENONE	GIANVI
ETHINYL ESTRADIOL/DROSPIRENONE	DROSPIRENONE-ETHINYL ESTRADIOL
ETHINYL ESTRADIOL/NORELGEST	ORTHO EVRA
ETHINYL ESTRADIOL/NORETH AC	FEMHRT
ETHYNODIOL D-ETHINYL ESTRADIOL	KELNOR 1-35
ETHYNODIOL D-ETHINYL ESTRADIOL	ZOVIA 1-50E
ETHYNODIOL D-ETHINYL ESTRADIOL	KELNOR 1/35
ETHYNODIOL D-ETHINYL ESTRADIOL	ZOVIA 1-35E
ETHYNODIOL D-ETHINYL ESTRADIOL	ZOVIA 1/35E
ETHYNODIOL D-ETHINYL ESTRADIOL	ZOVIA 1/50E
ETONOGESTREL/ETHINYL ESTRADIOL	NUVARING
L-NORGEST-ETH ESTR/ETHIN ESTRA	LOSEASONIQUE
L-NORGEST-ETH ESTR/ETHIN ESTRA	CAMRESE LO
L-NORGEST-ETH ESTR/ETHIN ESTRA	AMETHIA
L-NORGEST-ETH ESTR/ETHIN ESTRA	DAYSEE
L-NORGEST-ETH ESTR/ETHIN ESTRA	SEASONIQUE
L-NORGEST-ETH ESTR/ETHIN ESTRA	CAMRESE
L-NORGEST-ETH ESTR/ETHIN ESTRA	AMETHIA LO
L-NORGEST/E.ESTRADIOL-E.ESTRAD	AMETHIA
L-NORGEST/E.ESTRADIOL-E.ESTRAD	CAMRESE
L-NORGEST/E.ESTRADIOL-E.ESTRAD	LEVONORG-ETH ESTRAD ETH ESTRAD
L-NORGEST/E.ESTRADIOL-E.ESTRAD	CAMRESE LO
L-NORGEST/E.ESTRADIOL-E.ESTRAD	ASHLYNA
L-NORGEST/E.ESTRADIOL-E.ESTRAD	DAYSEE
L-NORGEST/E.ESTRADIOL-E.ESTRAD	QUARTETTE
L-NORGEST/E.ESTRADIOL-E.ESTRAD	AMETHIA LO
L-NORGEST/E.ESTRADION-E.ESTRAD	CAMRESE LO

The Journal of Law, Medicine & Ethics, 50 (2022): A1-A44. © 2022 The Author(s)

GENERIC NAME	BRAND NAME
L-NORGEST/E.ESTRADION-E.ESTRAD	CAMRESE
L-NORGEST/E.ESTRADION-E.ESTRAD	AMETHIA
L-NORGEST/E.ESTRADION-E.ESTRAD	ASHLYNA
L-NORGEST/E.ESTRADION-E.ESTRAD	DAYSEE
LEVONORGESTREL-ETH ESTRA	TRIPHASIL-28
LEVONORGESTREL-ETH ESTRA	JOLESSA
LEVONORGESTREL-ETH ESTRA	ENPRESSE
LEVONORGESTREL-ETH ESTRA	LYBREL
LEVONORGESTREL-ETH ESTRA	ALESSE-28
LEVONORGESTREL-ETH ESTRA	TRIVORA-28
LEVONORGESTREL-ETH ESTRA	INTROVALE
LEVONORGESTREL-ETH ESTRA	AMETHYST
LEVONORGESTREL-ETH ESTRA	PORTIA
LEVONORGESTREL-ETH ESTRA	LEVORA-28
LEVONORGESTREL-ETH ESTRA	LUTERA
LEVONORGESTREL-ETH ESTRA	SEASONALE
LEVONORGESTREL-ETH ESTRA	AVIANE
LEVONORGESTREL-ETH ESTRA	TRIPHASIL-21
LEVONORGESTREL-ETH ESTRA	QUASENSE
LEVONORGESTREL-ETH ESTRADIOL	PORTIA
LEVONORGESTREL-ETH ESTRADIOL	JOLESSA
LEVONORGESTREL-ETH ESTRADIOL	ENPRESSE
LEVONORGESTREL-ETH ESTRADIOL	LEVORA-28
LEVONORGESTREL-ETH ESTRADIOL	ORSYTHIA
LEVONORGESTREL-ETH ESTRADIOL	SRONYX
LEVONORGESTREL-ETH ESTRADIOL	LESSINA
LEVONORGESTREL-ETH ESTRADIOL	LEVONORGESTREL-ETH ESTRADIOL
LEVONORGESTREL-ETH ESTRADIOL	AVIANE
LEVONORGESTREL-ETH ESTRADIOL	INTROVALE
LEVONORGESTREL-ETH ESTRADIOL	QUASENSE
LEVONORGESTREL-ETH ESTRADIOL	ALTAVERA
LEVONORGESTREL-ETH ESTRADIOL	AMETHYST
LEVONORGESTREL-ETH ESTRADIOL	LUTERA

Table A.1 (continued)
Codes for Gender-Affirming Hormone Therapy

GENERIC NAME	BRAND NAME
LEVONORGESTREL-ETHIN ESTRADIOL	JOLESSA
LEVONORGESTREL-ETHIN ESTRADIOL	SETLAKIN
LEVONORGESTREL-ETHIN ESTRADIOL	LEVORA-28
LEVONORGESTREL-ETHIN ESTRADIOL	LUTERA
LEVONORGESTREL-ETHIN ESTRADIOL	LESSINA
LEVONORGESTREL-ETHIN ESTRADIOL	QUASENSE
LEVONORGESTREL-ETHIN ESTRADIOL	TRIVORA-28
LEVONORGESTREL-ETHIN ESTRADIOL	ALTAVERA
LEVONORGESTREL-ETHIN ESTRADIOL	CHATEAL
LEVONORGESTREL-ETHIN ESTRADIOL	INTROVALE
LEVONORGESTREL-ETHIN ESTRADIOL	PORTIA
LEVONORGESTREL-ETHIN ESTRADIOL	MARLISSA
LEVONORGESTREL-ETHIN ESTRADIOL	FALMINA
LEVONORGESTREL-ETHIN ESTRADIOL	AUBRA
LEVONORGESTREL-ETHIN ESTRADIOL	LARISSIA
LEVONORGESTREL-ETHIN ESTRADIOL	AVIANE
LEVONORGESTREL-ETHIN ESTRADIOL	LILLOW
LEVONORGESTREL-ETHIN ESTRADIOL	AMETHYST
LEVONORGESTREL-ETHIN ESTRADIOL	KURVELO
LEVONORGESTREL-ETHIN ESTRADIOL	VIENVA
LEVONORGESTREL-ETHIN ESTRADIOL	LEVONORGESTREL-ETH ESTRADIOL
LEVONORGESTREL-ETHIN ESTRADIOL	ORSYTHIA
LEVONORGESTREL-ETHIN ESTRADIOL	SRONYX
MEDROXYPROGESTERONE ACET	DEPO-PROVERA
MEDROXYPROGESTERONE ACET	PROVERA
MEDROXYPROGESTERONE ACET	CYCRIN
MEDROXYPROGESTERONE ACET	MEDROXYPROGESTERONE ACETATE
MEDROXYPROGESTERONE ACETATE	PROVERA
MEDROXYPROGESTERONE ACETATE	MEDROXYPROGESTERONE ACETATE
MEDROXYPROGESTERONE ACETATE	DEPO-PROVERA
MEDROXYPROGESTERONE ACETATE	DEPO-SUBQ PROVERA 104
NORELGESTROMIN/ETHIN.ESTRADIOL	ORTHO EVRA
NORELGESTROMIN/ETHIN.ESTRADIOL	XULANE

GENERIC NAME	BRAND NAME
NORETH A-ET ESTRA/FE FUMARATE	GILDESS FE
NORETH A-ET ESTRA/FE FUMARATE	ESTROSTEP FE
NORETH A-ET ESTRA/FE FUMARATE	JUNEL FE
NORETH A-ET ESTRA/FE FUMARATE	LOESTRIN FE
NORETH A-ET ESTRA/FE FUMARATE	LO LOESTRIN FE
NORETH A-ET ESTRA/FE FUMARATE	MICROGESTIN FE
NORETH A-ET ESTRA/FE FUMARATE	LOESTRIN 24 FE
NORETH A-ET ESTRA/FE FUMARATE	TILIA FE
NORETH-ETHINYL ESTRADIOL/IRON	FEMCON FE
NORETH-ETHINYL ESTRADIOL/IRON	ZEOSA
NORETH-ETHINYL ESTRADIOL/IRON	ZENCHENT FE
NORETH-ETHINYL ESTRADIOL/IRON	GENERESS FE
NORETHIND AC/ETHINYL ESTRADIOL	FEMHRT
NORETHIND AC/ETHINYL ESTRADIOL	JINTELI
NORETHIND AC/ETHINYL ESTRADIOL	JEVANTIQUE
NORETHINDRONE A-E ESTRADIOL	MICROGESTIN
NORETHINDRONE A-E ESTRADIOL	GILDESS
NORETHINDRONE A-E ESTRADIOL	LOESTRIN
NORETHINDRONE A-E ESTRADIOL	JUNEL
NORETHINDRONE AC-ETH ESTRADIOL	JUNEL
NORETHINDRONE AC-ETH ESTRADIOL	JINTELI
NORETHINDRONE AC-ETH ESTRADIOL	NORETHINDRON-ETHINYL ESTRADIOL
NORETHINDRONE AC-ETH ESTRADIOL	LARIN
NORETHINDRONE AC-ETH ESTRADIOL	GILDESS
NORETHINDRONE AC-ETH ESTRADIOL	MICROGESTIN
NORETHINDRONE AC-ETH ESTRADIOL	FYAVOLV
NORETHINDRONE AC-ETH ESTRADIOL	FEMHRT
NORETHINDRONE-E.ESTRADIOL-IRON	HAILEY 24 FE
NORETHINDRONE-E.ESTRADIOL-IRON	BLISOVI FE
NORETHINDRONE-E.ESTRADIOL-IRON	JUNEL FE 24
NORETHINDRONE-E.ESTRADIOL-IRON	MICROGESTIN FE
NORETHINDRONE-E.ESTRADIOL-IRON	TAYTULLA
NORETHINDRONE-E.ESTRADIOL-IRON	BLISOVI 24 FE

The Journal of Law, Medicine & Ethics, 50 (2022): A1-A44. © 2022 The Author(s)

Table A.1 (continued)
Codes for Gender-Affirming Hormone Therapy

GENERIC NAME	BRAND NAME
NORETHINDRONE-E.ESTRADIOL-IRON	GILDESS 24 FE
NORETHINDRONE-E.ESTRADIOL-IRON	MINASTRIN 24 FE
NORETHINDRONE-E.ESTRADIOL-IRON	LOMEDIA 24 FE
NORETHINDRONE-E.ESTRADIOL-IRON	LO LOESTRIN FE
NORETHINDRONE-E.ESTRADIOL-IRON	AUROVELA FE
NORETHINDRONE-E.ESTRADIOL-IRON	GILDESS FE
NORETHINDRONE-E.ESTRADIOL-IRON	NORETHIN-ETH ESTRA-FERROUS FUM
NORETHINDRONE-E.ESTRADIOL-IRON	LARIN FE
NORETHINDRONE-E.ESTRADIOL-IRON	LARIN 24 FE
NORETHINDRONE-E.ESTRADIOL-IRON	NORETHIN-ETH ESTRA FERROUS FUM
NORETHINDRONE-E.ESTRADIOL-IRON	JUNEL FE
NORETHINDRONE-ETHIN. ESTRADIOL	NORTREL
NORETHINDRONE-ETHIN. ESTRADIOL	DASETTA
NORETHINDRONE-ETHINYL ESTRAD	DASETTA
NORETHINDRONE-ETHINYL ESTRAD	PIRMELLA
NORETHINDRONE-ETHINYL ESTRAD	WERA
NORETHINDRONE-ETHINYL ESTRAD	GILDAGIA
NORETHINDRONE-ETHINYL ESTRAD	NECON
NORETHINDRONE-ETHINYL ESTRAD	OVCON-35
NORETHINDRONE-ETHINYL ESTRAD	BRIELLYN
NORETHINDRONE-ETHINYL ESTRAD	PHILITH
NORETHINDRONE-ETHINYL ESTRAD	ORTHO-NOVUM
NORETHINDRONE-ETHINYL ESTRAD	VYFEMLA
NORETHINDRONE-ETHINYL ESTRAD	ZENCHENT
NORETHINDRONE-ETHINYL ESTRAD	LEENA
NORETHINDRONE-ETHINYL ESTRAD	NORTREL
NORETHINDRONE-ETHINYL ESTRAD	CYCLAFEM
NORETHINDRONE-ETHINYL ESTRAD	ALYACEN
NORETHINDRONE-ETHINYL ESTRAD	BALZIVA
NORETHINDRONE-ETHINYL ESTRAD	MODICON
NORETHINDRONE-MESTRANOL	ORTHO-NOVUM
NORETHINDRONE-MESTRANOL	NECON
NORGESTIMATE-ETHINYL ESTRADIOL	FEMYNOR

The Journal of Law, Medicine & Ethics, 50 (2022): A1-A44. © 2022 The Author(s)

GENERIC NAME	BRAND NAME
NORGESTIMATE-ETHINYL ESTRADIOL	TRI-SPRINTEC
NORGESTIMATE-ETHINYL ESTRADIOL	MONONESSA
NORGESTIMATE-ETHINYL ESTRADIOL	TRI-LO-ESTARYLLA
NORGESTIMATE-ETHINYL ESTRADIOL	TRI-LO-MARZIA
NORGESTIMATE-ETHINYL ESTRADIOL	ORTHO-CYCLEN
NORGESTIMATE-ETHINYL ESTRADIOL	TRI-LO-SPRINTEC
NORGESTIMATE-ETHINYL ESTRADIOL	MONO-LINYAH
NORGESTIMATE-ETHINYL ESTRADIOL	MILI
NORGESTIMATE-ETHINYL ESTRADIOL	ORTHO TRI-CYCLEN LO
NORGESTIMATE-ETHINYL ESTRADIOL	TRI-PREVIFEM
NORGESTIMATE-ETHINYL ESTRADIOL	TRINESSA
NORGESTIMATE-ETHINYL ESTRADIOL	PREVIFEM
NORGESTIMATE-ETHINYL ESTRADIOL	SPRINTEC
NORGESTIMATE-ETHINYL ESTRADIOL	TRI-VYLIBRA
NORGESTIMATE-ETHINYL ESTRADIOL	TRINESSA LO
NORGESTIMATE-ETHINYL ESTRADIOL	TRI-ESTARYLLA
NORGESTIMATE-ETHINYL ESTRADIOL	TRI-LINYAH
NORGESTIMATE-ETHINYL ESTRADIOL	ESTARYLLA
NORGESTIMATE-ETHINYL ESTRADIOL	NORGESTIMATE-ETHINYL ESTRADIOL
NORGESTIMATE-ETHINYL ESTRADIOL	ORTHO TRI-CYCLEN
NORGESTIMATE-ETHINYL ESTRADIOL	TRI FEMYNOR
NORGESTREL-ETHINYL ESTRADIOL	LO/OVRAL-21
NORGESTREL-ETHINYL ESTRADIOL	ELINEST
NORGESTREL-ETHINYL ESTRADIOL	LOW-OGESTREL
NORGESTREL-ETHINYL ESTRADIOL	CRYSELLE
NORGESTREL-ETHINYL ESTRADIOL	OGESTREL
PROGESTERONE	PROGESTERONE IN OIL
PROGESTERONE	PROGESTERONE
SPIRONOLACT/HYDROCHLOROTHIAZID	SPIRONOLACTONE-HCTZ
SPIRONOLACT/HYDROCHLOROTHIAZID	ALDACTAZIDE
SPIRONOLACTONE	SPIRONOLACTONE
SPIRONOLACTONE	ALDACTONE
SPIRONOLACTONE/HCTZ	SPIRONOLACTONE W/HCTZ

Table A.1 (continued)
Codes for Gender-Affirming Hormone Therapy

GENERIC NAME	BRAND NAME
SPIRONOLACTONE/HCTZ	ALDACTAZIDE
GnRH Analogs	
ELAGOLIX SODIUM	ORILISSA
LEUPROLIDE ACETATE	LUPRON DEPOT-PED
LEUPROLIDE ACETATE	ELIGARD
LEUPROLIDE ACETATE	LEUPROLIDE ACETATE
LEUPROLIDE ACETATE	LUPRON DEPOT
LEUPROLIDE ACETATE	LUPRON
NAFARELIN ACETATE	SYNAREL

The Journal of Law, Medicine & Ethics, 50 (2022): A1-A44. © 2022 The Author(s)

CODE	TYPE	DESCRIPTION	CATEGORY
831	ICD-9	Repair of blepharoptosis by frontalis muscle technique with suture	Facial Feminization
832	ICD-9	Repair of blepharoptosis by frontalis muscle technique with fascial sling	Facial Feminization
833	ICD-9	Repair of blepharoptosis by resection or advancement of levator muscle or aponeurosis	Facial Feminization
834	ICD-9	Repair of blepharoptosis by other levator muscle techniques	Facial Feminization
835	ICD-9	Repair of blepharoptosis by tarsal technique	Facial Feminization
836	ICD-9	Repair of blepharoptosis by other techniques	Facial Feminization
7646	ICD-9	Other reconstruction of other facial bone	Facial Feminization
7667	ICD-9	Reduction genioplasty	Facial Feminization
7668	ICD-9	Augmentation genioplasty	Facial Feminization
7669	ICD-9	Other facial bone repair	Facial Feminization
7691	ICD-9	Bone graft to facial bone	Facial Feminization
7692	ICD-9	Insertion of synthetic implant in facial bone	Facial Feminization
080N0ZZ	ICD-10	Alteration of Right Upper Eyelid, Open Approach	Facial Feminization
080P0ZZ	ICD-10	Alteration of Left Upper Eyelid, Open Approach	Facial Feminization
08SN0ZZ	ICD-10	Reposition Right Upper Eyelid, Open Approach	Facial Feminization
08SN3ZZ	ICD-10	Reposition Right Upper Eyelid, Percutaneous Approach	Facial Feminization
08SNXZZ	ICD-10	Reposition Right Upper Eyelid, External Approach	Facial Feminization
08SP0ZZ	ICD-10	Reposition Left Upper Eyelid, Open Approach	Facial Feminization
08SP3ZZ	ICD-10	Reposition Left Upper Eyelid, Percutaneous Approach	Facial Feminization
08SPXZZ	ICD-10	Reposition Left Upper Eyelid, External Approach	Facial Feminization
08SQ0ZZ	ICD-10	Reposition Right Lower Eyelid, Open Approach	Facial Feminization
08SQ3ZZ	ICD-10	Reposition Right Lower Eyelid, Percutaneous Approach	Facial Feminization
08SQXZZ	ICD-10	Reposition Right Lower Eyelid, External Approach	Facial Feminization
08SR0ZZ	ICD-10	Reposition Left Lower Eyelid, Open Approach	Facial Feminization
08SR3ZZ	ICD-10	Reposition Left Lower Eyelid, Percutaneous Approach	Facial Feminization
08SRXZZ	ICD-10	Reposition Left Lower Eyelid, External Approach	Facial Feminization
090K07Z	ICD-10	Alteration of Nasal Mucosa and Soft Tissue with Autologous Tissue Substitute, Open Approach	Facial Feminization
090K0ZZ	ICD-10	Alteration of Nasal Mucosa and Soft Tissue, Open Approach	Facial Feminization
090K37Z	ICD-10	Alteration of Nasal Mucosa and Soft Tissue with Autologous Tissue Substitute, Percutaneous Approach	Facial Feminization
090K3ZZ	ICD-10	Alteration of Nasal Mucosa and Soft Tissue, Percutaneous Approach	Facial Feminization
09BM0ZZ	ICD-10	Excision of Nasal Septum, Open Approach	Facial Feminization
09SK0ZZ	ICD-10	Reposition Nasal Mucosa and Soft Tissue, Open Approach	Facial Feminization
0BB10ZZ	ICD-10	Excision of Trachea, Open Approach	Facial Feminization
0CU137Z	ICD-10	Supplement Lower Lip with Autologous Tissue Substitute, Percutaneous Approach	Facial Feminization
0HB1XZZ	ICD-10	Excision of Face Skin, External Approach	Facial Feminization
0HD0XZZ	ICD-10	Extraction of Scalp Skin, External Approach	Facial Feminization
0HD1XZZ	ICD-10	Extraction of Face Skin, External Approach	Facial Feminization
0HD4XZZ	ICD-10	Extraction of Neck Skin, External Approach	Facial Feminization
0HX0XZZ	ICD-10	Transfer Scalp Skin, External Approach	Facial Feminization
0HX1XZZ	ICD-10	Transfer Face Skin, External Approach	Facial Feminization
0J013ZZ	ICD-10	Alteration of Face Subcutaneous Tissue and Fascia, Percutaneous Approach	Facial Feminization
0J043ZZ	ICD-10	Alteration of Right Neck Subcutaneous Tissue and Fascia, Percutaneous Approach	Facial Feminization
0J053ZZ	ICD-10	Alteration of Left Neck Subcutaneous Tissue and Fascia, Percutaneous Approach	Facial Feminization
0JC10ZZ	ICD-10	Extirpation of Matter from Face Subcutaneous Tissue and Fascia, Open Approach	Facial Feminization
0JX00ZB	ICD-10	Transfer Scalp Subcutaneous Tissue and Fascia with Skin and Subcutaneous Tissue, Open Approach	Facial Feminization

0KB10ZZ	ICD-10	Excision of Facial Muscle, Open Approach	Facial Feminization
0KS10ZZ	ICD-10	Reposition Facial Muscle, Open Approach	Facial Feminization
0KS14ZZ	ICD-10	Reposition Facial Muscle, Percutaneous Endoscopic Approach	Facial Feminization
0NB10ZZ	ICD-10	Excision of Frontal Bone, Open Approach	Facial Feminization
0NBT0ZZ	ICD-10	Excision of Right Mandible, Open Approach	Facial Feminization
0NBT3ZZ	ICD-10	Excision of Right Mandible, Percutaneous Approach	Facial Feminization
0NBV0ZZ	ICD-10	Excision of Left Mandible, Open Approach	Facial Feminization
0NBV3ZZ	ICD-10	Excision of Left Mandible, Percutaneous Approach	Facial Feminization
0NNC0ZZ	ICD-10	Release Right Sphenoid Bone, Open Approach	Facial Feminization
0NNC3ZZ	ICD-10	Release Right Sphenoid Bone, Percutaneous Approach	Facial Feminization
0NNC4ZZ	ICD-10	Release Right Sphenoid Bone, Percutaneous Endoscopic Approach	Facial Feminization
0NND0ZZ	ICD-10	Release Left Sphenoid Bone, Open Approach	Facial Feminization
0NND3ZZ	ICD-10	Release Left Sphenoid Bone, Percutaneous Approach	Facial Feminization
0NND4ZZ	ICD-10	Release Left Sphenoid Bone, Percutaneous Endoscopic Approach	Facial Feminization
0NNF0ZZ	ICD-10	Release Right Ethmoid Bone, Open Approach	Facial Feminization
0NNF3ZZ	ICD-10	Release Right Ethmoid Bone, Percutaneous Approach	Facial Feminization
0NNF4ZZ	ICD-10	Release Right Ethmoid Bone, Percutaneous Endoscopic Approach	Facial Feminization
0NNG0ZZ	ICD-10	Release Left Ethmoid Bone, Open Approach	Facial Feminization
0NNG3ZZ	ICD-10	Release Left Ethmoid Bone, Percutaneous Approach	Facial Feminization
0NNG4ZZ	ICD-10	Release Left Ethmoid Bone, Percutaneous Endoscopic Approach	Facial Feminization
0NNH0ZZ	ICD-10	Release Right Lacrimal Bone, Open Approach	Facial Feminization
0NNH3ZZ	ICD-10	Release Right Lacrimal Bone, Percutaneous Approach	Facial Feminization
0NNH4ZZ	ICD-10	Release Right Lacrimal Bone, Percutaneous Endoscopic Approach	Facial Feminization
0NNJ0ZZ	ICD-10	Release Left Lacrimal Bone, Open Approach	Facial Feminization
0NNJ3ZZ	ICD-10	Release Left Lacrimal Bone, Percutaneous Approach	Facial Feminization
0NNJ4ZZ	ICD-10	Release Left Lacrimal Bone, Percutaneous Endoscopic Approach	Facial Feminization
0NNK0ZZ	ICD-10	Release Right Palatine Bone, Open Approach	Facial Feminization
0NNK3ZZ	ICD-10	Release Right Palatine Bone, Percutaneous Approach	Facial Feminization
0NNK4ZZ	ICD-10	Release Right Palatine Bone, Percutaneous Endoscopic Approach	Facial Feminization
0NNL0ZZ	ICD-10	Release Left Palatine Bone, Open Approach	Facial Feminization
0NNL3ZZ	ICD-10	Release Left Palatine Bone, Percutaneous Approach	Facial Feminization
0NNL4ZZ	ICD-10	Release Left Palatine Bone, Percutaneous Endoscopic Approach	Facial Feminization
0NNM0ZZ	ICD-10	Release Right Zygomatic Bone, Open Approach	Facial Feminization
0NNM3ZZ	ICD-10	Release Right Zygomatic Bone, Percutaneous Approach	Facial Feminization
0NNM4ZZ	ICD-10	Release Right Zygomatic Bone, Percutaneous Endoscopic Approach	Facial Feminization
0NNN0ZZ	ICD-10	Release Left Zygomatic Bone, Open Approach	Facial Feminization
0NNN3ZZ	ICD-10	Release Left Zygomatic Bone, Percutaneous Approach	Facial Feminization
0NNN4ZZ	ICD-10	Release Left Zygomatic Bone, Percutaneous Endoscopic Approach	Facial Feminization
0NNP0ZZ	ICD-10	Release Right Orbit, Open Approach	Facial Feminization
0NNP3ZZ	ICD-10	Release Right Orbit, Percutaneous Approach	Facial Feminization
0NNP4ZZ	ICD-10	Release Right Orbit, Percutaneous Endoscopic Approach	Facial Feminization
0NNQ0ZZ	ICD-10	Release Left Orbit, Open Approach	Facial Feminization
0NNQ3ZZ	ICD-10	Release Left Orbit, Percutaneous Approach	Facial Feminization
0NNQ4ZZ	ICD-10	Release Left Orbit, Percutaneous Endoscopic Approach	Facial Feminization
0NNR0ZZ	ICD-10	Release Right Maxilla, Open Approach	Facial Feminization
0NNR3ZZ	ICD-10	Release Right Maxilla, Percutaneous Approach	Facial Feminization

0NNR4ZZ	ICD-10	Release Right Maxilla, Percutaneous Endoscopic Approach	Facial Feminization
0NNS0ZZ	ICD-10	Release Left Maxilla, Open Approach	Facial Feminization
0NNS3ZZ	ICD-10	Release Left Maxilla, Percutaneous Approach	Facial Feminization
0NNS4ZZ	ICD-10	Release Left Maxilla, Percutaneous Endoscopic Approach	Facial Feminization
0NNT0ZZ	ICD-10	Release Right Mandible, Open Approach	Facial Feminization
0NNT3ZZ	ICD-10	Release Right Mandible, Percutaneous Approach	Facial Feminization
0NNT4ZZ	ICD-10	Release Right Mandible, Percutaneous Endoscopic Approach	Facial Feminization
0NNV0ZZ	ICD-10	Release Left Mandible, Open Approach	Facial Feminization
0NNV3ZZ	ICD-10	Release Left Mandible, Percutaneous Approach	Facial Feminization
0NNV4ZZ	ICD-10	Release Left Mandible, Percutaneous Endoscopic Approach	Facial Feminization
0NPW04Z	ICD-10	Removal of Internal Fixation Device from Facial Bone, Open Approach	Facial Feminization
0NQC0ZZ	ICD-10	Repair Right Sphenoid Bone, Open Approach	Facial Feminization
0NQC3ZZ	ICD-10	Repair Right Sphenoid Bone, Percutaneous Approach	Facial Feminization
0NQC4ZZ	ICD-10	Repair Right Sphenoid Bone, Percutaneous Endoscopic Approach	Facial Feminization
0NQCXZZ	ICD-10	Repair Right Sphenoid Bone, External Approach	Facial Feminization
0NQD0ZZ	ICD-10	Repair Left Sphenoid Bone, Open Approach	Facial Feminization
0NQD3ZZ	ICD-10	Repair Left Sphenoid Bone, Percutaneous Approach	Facial Feminization
0NQD4ZZ	ICD-10	Repair Left Sphenoid Bone, Percutaneous Endoscopic Approach	Facial Feminization
0NQDXZZ	ICD-10	Repair Left Sphenoid Bone, External Approach	Facial Feminization
0NQF0ZZ	ICD-10	Repair Right Ethmoid Bone, Open Approach	Facial Feminization
0NQF3ZZ	ICD-10	Repair Right Ethmoid Bone, Percutaneous Approach	Facial Feminization
0NQF4ZZ	ICD-10	Repair Right Ethmoid Bone, Percutaneous Endoscopic Approach	Facial Feminization
0NQFXZZ	ICD-10	Repair Right Ethmoid Bone, External Approach	Facial Feminization
0NQG0ZZ	ICD-10	Repair Left Ethmoid Bone, Open Approach	Facial Feminization
0NQG3ZZ	ICD-10	Repair Left Ethmoid Bone, Percutaneous Approach	Facial Feminization
0NQG4ZZ	ICD-10	Repair Left Ethmoid Bone, Percutaneous Endoscopic Approach	Facial Feminization
0NQGXZZ	ICD-10	Repair Left Ethmoid Bone, External Approach	Facial Feminization
0NQH0ZZ	ICD-10	Repair Right Lacrimal Bone, Open Approach	Facial Feminization
0NQH3ZZ	ICD-10	Repair Right Lacrimal Bone, Percutaneous Approach	Facial Feminization
0NQH4ZZ	ICD-10	Repair Right Lacrimal Bone, Percutaneous Endoscopic Approach	Facial Feminization
0NQHXZZ	ICD-10	Repair Right Lacrimal Bone, External Approach	Facial Feminization
0NQJ0ZZ	ICD-10	Repair Left Lacrimal Bone, Open Approach	Facial Feminization
0NQJ3ZZ	ICD-10	Repair Left Lacrimal Bone, Percutaneous Approach	Facial Feminization
0NQJ4ZZ	ICD-10	Repair Left Lacrimal Bone, Percutaneous Endoscopic Approach	Facial Feminization
0NQJXZZ	ICD-10	Repair Left Lacrimal Bone, External Approach	Facial Feminization
0NQK0ZZ	ICD-10	Repair Right Palatine Bone, Open Approach	Facial Feminization
0NQK3ZZ	ICD-10	Repair Right Palatine Bone, Percutaneous Approach	Facial Feminization
0NQK4ZZ	ICD-10	Repair Right Palatine Bone, Percutaneous Endoscopic Approach	Facial Feminization
0NQKXZZ	ICD-10	Repair Right Palatine Bone, External Approach	Facial Feminization
0NQL0ZZ	ICD-10	Repair Left Palatine Bone, Open Approach	Facial Feminization
0NQL3ZZ	ICD-10	Repair Left Palatine Bone, Percutaneous Approach	Facial Feminization
0NQL4ZZ	ICD-10	Repair Left Palatine Bone, Percutaneous Endoscopic Approach	Facial Feminization
0NQLXZZ	ICD-10	Repair Left Palatine Bone, External Approach	Facial Feminization
0NQM0ZZ	ICD-10	Repair Right Zygomatic Bone, Open Approach	Facial Feminization
0NQM3ZZ	ICD-10	Repair Right Zygomatic Bone, Percutaneous Approach	Facial Feminization
0NQM4ZZ	ICD-10	Repair Right Zygomatic Bone, Percutaneous Endoscopic Approach	Facial Feminization

0NQMXZZ	ICD-10	Repair Right Zygomatic Bone, External Approach	Facial Feminization
0NQN0ZZ	ICD-10	Repair Left Zygomatic Bone, Open Approach	Facial Feminization
0NQN3ZZ	ICD-10	Repair Left Zygomatic Bone, Percutaneous Approach	Facial Feminization
0NQN4ZZ	ICD-10	Repair Left Zygomatic Bone, Percutaneous Endoscopic Approach	Facial Feminization
0NQNXZZ	ICD-10	Repair Left Zygomatic Bone, External Approach	Facial Feminization
0NQX0ZZ	ICD-10	Repair Hyoid Bone, Open Approach	Facial Feminization
0NQX3ZZ	ICD-10	Repair Hyoid Bone, Percutaneous Approach	Facial Feminization
0NQX4ZZ	ICD-10	Repair Hyoid Bone, Percutaneous Endoscopic Approach	Facial Feminization
0NQXXZZ	ICD-10	Repair Hyoid Bone, External Approach	Facial Feminization
0NRC07Z	ICD-10	Replacement of Right Sphenoid Bone with Autologous Tissue Substitute, Open Approach	Facial Feminization
0NRC0JZ	ICD-10	Replacement of Right Sphenoid Bone with Synthetic Substitute, Open Approach	Facial Feminization
0NRC0KZ	ICD-10	Replacement of Right Sphenoid Bone with Nonautologous Tissue Substitute, Open Approach	Facial Feminization
0NRC37Z	ICD-10	Replacement of Right Sphenoid Bone with Autologous Tissue Substitute, Percutaneous Approach	Facial Feminization
0NRC3JZ	ICD-10	Replacement of Right Sphenoid Bone with Synthetic Substitute, Percutaneous Approach	Facial Feminization
0NRC3KZ	ICD-10	Replacement of Right Sphenoid Bone with Nonautologous Tissue Substitute, Percutaneous Approach	Facial Feminization
0NRC47Z	ICD-10	Replacement of Right Sphenoid Bone with Autologous Tissue Substitute, Percutaneous Endoscopic Approach	Facial Feminization
0NRC4JZ	ICD-10	Replacement of Right Sphenoid Bone with Synthetic Substitute, Percutaneous Endoscopic Approach	Facial Feminization
0NRC4KZ	ICD-10	Replacement of Right Sphenoid Bone with Nonautologous Tissue Substitute, Percutaneous Endoscopic Approach	Facial Feminization
0NRD07Z	ICD-10	Replacement of Left Sphenoid Bone with Autologous Tissue Substitute, Open Approach	Facial Feminization
0NRD0JZ	ICD-10	Replacement of Left Sphenoid Bone with Synthetic Substitute, Open Approach	Facial Feminization
0NRD0KZ	ICD-10	Replacement of Left Sphenoid Bone with Nonautologous Tissue Substitute, Open Approach	Facial Feminization
0NRD37Z	ICD-10	Replacement of Left Sphenoid Bone with Autologous Tissue Substitute, Percutaneous Approach	Facial Feminization
0NRD3JZ	ICD-10	Replacement of Left Sphenoid Bone with Synthetic Substitute, Percutaneous Approach	Facial Feminization
0NRD3KZ	ICD-10	Replacement of Left Sphenoid Bone with Nonautologous Tissue Substitute, Percutaneous Approach	Facial Feminization
0NRD47Z	ICD-10	Replacement of Left Sphenoid Bone with Autologous Tissue Substitute, Percutaneous Endoscopic Approach	Facial Feminization
0NRD4JZ	ICD-10	Replacement of Left Sphenoid Bone with Synthetic Substitute, Percutaneous Endoscopic Approach	Facial Feminization
0NRD4KZ	ICD-10	Replacement of Left Sphenoid Bone with Nonautologous Tissue Substitute, Percutaneous Endoscopic Approach	Facial Feminization
0NRF07Z	ICD-10	Replacement of Right Ethmoid Bone with Autologous Tissue Substitute, Open Approach	Facial Feminization
0NRF0JZ	ICD-10	Replacement of Right Ethmoid Bone with Synthetic Substitute, Open Approach	Facial Feminization
0NRF0KZ	ICD-10	Replacement of Right Ethmoid Bone with Nonautologous Tissue Substitute, Open Approach	Facial Feminization
0NRF37Z	ICD-10	Replacement of Right Ethmoid Bone with Autologous Tissue Substitute, Percutaneous Approach	Facial Feminization
0NRF3JZ	ICD-10	Replacement of Right Ethmoid Bone with Synthetic Substitute, Percutaneous Approach	Facial Feminization
0NRF3KZ	ICD-10	Replacement of Right Ethmoid Bone with Nonautologous Tissue Substitute, Percutaneous Approach	Facial Feminization
0NRF47Z	ICD-10	Replacement of Right Ethmoid Bone with Autologous Tissue Substitute, Percutaneous Endoscopic Approach	Facial Feminization
0NRF4JZ	ICD-10	Replacement of Right Ethmoid Bone with Synthetic Substitute, Percutaneous Endoscopic Approach	Facial Feminization
0NRF4KZ	ICD-10	Replacement of Right Ethmoid Bone with Nonautologous Tissue Substitute, Percutaneous Endoscopic Approach	Facial Feminization
0NRG07Z	ICD-10	Replacement of Left Ethmoid Bone with Autologous Tissue Substitute, Open Approach	Facial Feminization
0NRG0JZ	ICD-10	Replacement of Left Ethmoid Bone with Synthetic Substitute, Open Approach	Facial Feminization
0NRG0KZ	ICD-10	Replacement of Left Ethmoid Bone with Nonautologous Tissue Substitute, Open Approach	Facial Feminization
0NRG37Z	ICD-10	Replacement of Left Ethmoid Bone with Autologous Tissue Substitute, Percutaneous Approach	Facial Feminization
0NRG3JZ	ICD-10	Replacement of Left Ethmoid Bone with Synthetic Substitute, Percutaneous Approach	Facial Feminization
0NRG3KZ	ICD-10	Replacement of Left Ethmoid Bone with Nonautologous Tissue Substitute, Percutaneous Approach	Facial Feminization
0NRG47Z	ICD-10	Replacement of Left Ethmoid Bone with Autologous Tissue Substitute, Percutaneous Endoscopic Approach	Facial Feminization
0NRG4JZ	ICD-10	Replacement of Left Ethmoid Bone with Synthetic Substitute, Percutaneous Endoscopic Approach	Facial Feminization
0NRG4KZ	ICD-10	Replacement of Left Ethmoid Bone with Nonautologous Tissue Substitute, Percutaneous Endoscopic Approach	Facial Feminization
0NRH07Z	ICD-10	Replacement of Right Lacrimal Bone with Autologous Tissue Substitute, Open Approach	Facial Feminization

0NRH0JZ	ICD-10	Replacement of Right Lacrimal Bone with Synthetic Substitute, Open Approach	Facial Feminization
0NRH0KZ	ICD-10	Replacement of Right Lacrimal Bone with Nonautologous Tissue Substitute, Open Approach	Facial Feminization
0NRH37Z	ICD-10	Replacement of Right Lacrimal Bone with Autologous Tissue Substitute, Percutaneous Approach	Facial Feminization
0NRH3JZ	ICD-10	Replacement of Right Lacrimal Bone with Synthetic Substitute, Percutaneous Approach	Facial Feminization
0NRH3KZ	ICD-10	Replacement of Right Lacrimal Bone with Nonautologous Tissue Substitute, Percutaneous Approach	Facial Feminization
0NRH47Z	ICD-10	Replacement of Right Lacrimal Bone with Autologous Tissue Substitute, Percutaneous Endoscopic Approach	Facial Feminization
0NRH4JZ	ICD-10	Replacement of Right Lacrimal Bone with Synthetic Substitute, Percutaneous Endoscopic Approach	Facial Feminization
0NRH4KZ	ICD-10	Replacement of Right Lacrimal Bone with Nonautologous Tissue Substitute, Percutaneous Endoscopic Approach	Facial Feminization
0NRJ07Z	ICD-10	Replacement of Left Lacrimal Bone with Autologous Tissue Substitute, Open Approach	Facial Feminization
0NRJ0JZ	ICD-10	Replacement of Left Lacrimal Bone with Synthetic Substitute, Open Approach	Facial Feminization
0NRJ0KZ	ICD-10	Replacement of Left Lacrimal Bone with Nonautologous Tissue Substitute, Open Approach	Facial Feminization
0NRJ37Z	ICD-10	Replacement of Left Lacrimal Bone with Autologous Tissue Substitute, Percutaneous Approach	Facial Feminization
0NRJ3JZ	ICD-10	Replacement of Left Lacrimal Bone with Synthetic Substitute, Percutaneous Approach	Facial Feminization
0NRJ3KZ	ICD-10	Replacement of Left Lacrimal Bone with Nonautologous Tissue Substitute, Percutaneous Approach	Facial Feminization
0NRJ47Z	ICD-10	Replacement of Left Lacrimal Bone with Autologous Tissue Substitute, Percutaneous Endoscopic Approach	Facial Feminization
0NRJ4JZ	ICD-10	Replacement of Left Lacrimal Bone with Synthetic Substitute, Percutaneous Endoscopic Approach	Facial Feminization
0NRJ4KZ	ICD-10	Replacement of Left Lacrimal Bone with Nonautologous Tissue Substitute, Percutaneous Endoscopic Approach	Facial Feminization
0NRK07Z	ICD-10	Replacement of Right Palatine Bone with Autologous Tissue Substitute, Open Approach	Facial Feminization
0NRK0JZ	ICD-10	Replacement of Right Palatine Bone with Synthetic Substitute, Open Approach	Facial Feminization
0NRK0KZ	ICD-10	Replacement of Right Palatine Bone with Nonautologous Tissue Substitute, Open Approach	Facial Feminization
0NRK37Z	ICD-10	Replacement of Right Palatine Bone with Autologous Tissue Substitute, Percutaneous Approach	Facial Feminization
0NRK3JZ	ICD-10	Replacement of Right Palatine Bone with Synthetic Substitute, Percutaneous Approach	Facial Feminization
0NRK3KZ	ICD-10	Replacement of Right Palatine Bone with Nonautologous Tissue Substitute, Percutaneous Approach	Facial Feminization
0NRK47Z	ICD-10	Replacement of Right Palatine Bone with Autologous Tissue Substitute, Percutaneous Endoscopic Approach	Facial Feminization
0NRK4JZ	ICD-10	Replacement of Right Palatine Bone with Synthetic Substitute, Percutaneous Endoscopic Approach	Facial Feminization
0NRK4KZ	ICD-10	Replacement of Right Palatine Bone with Nonautologous Tissue Substitute, Percutaneous Endoscopic Approach	Facial Feminization
0NRL07Z	ICD-10	Replacement of Left Palatine Bone with Autologous Tissue Substitute, Open Approach	Facial Feminization
0NRL0JZ	ICD-10	Replacement of Left Palatine Bone with Synthetic Substitute, Open Approach	Facial Feminization
0NRL0KZ	ICD-10	Replacement of Left Palatine Bone with Nonautologous Tissue Substitute, Open Approach	Facial Feminization
0NRL37Z	ICD-10	Replacement of Left Palatine Bone with Autologous Tissue Substitute, Percutaneous Approach	Facial Feminization
0NRL3JZ	ICD-10	Replacement of Left Palatine Bone with Synthetic Substitute, Percutaneous Approach	Facial Feminization
0NRL3KZ	ICD-10	Replacement of Left Palatine Bone with Nonautologous Tissue Substitute, Percutaneous Approach	Facial Feminization
0NRL47Z	ICD-10	Replacement of Left Palatine Bone with Autologous Tissue Substitute, Percutaneous Endoscopic Approach	Facial Feminization
0NRL4JZ	ICD-10	Replacement of Left Palatine Bone with Synthetic Substitute, Percutaneous Endoscopic Approach	Facial Feminization
0NRL4KZ	ICD-10	Replacement of Left Palatine Bone with Nonautologous Tissue Substitute, Percutaneous Endoscopic Approach	Facial Feminization
0NRM07Z	ICD-10	Replacement of Right Zygomatic Bone with Autologous Tissue Substitute, Open Approach	Facial Feminization
0NRM0JZ	ICD-10	Replacement of Right Zygomatic Bone with Synthetic Substitute, Open Approach	Facial Feminization
0NRM0KZ	ICD-10	Replacement of Right Zygomatic Bone with Nonautologous Tissue Substitute, Open Approach	Facial Feminization
0NRM37Z	ICD-10	Replacement of Right Zygomatic Bone with Autologous Tissue Substitute, Percutaneous Approach	Facial Feminization
0NRM3JZ	ICD-10	Replacement of Right Zygomatic Bone with Synthetic Substitute, Percutaneous Approach	Facial Feminization
0NRM3KZ	ICD-10	Replacement of Right Zygomatic Bone with Nonautologous Tissue Substitute, Percutaneous Approach	Facial Feminization
0NRM47Z	ICD-10	Replacement of Right Zygomatic Bone with Autologous Tissue Substitute, Percutaneous Endoscopic Approach	Facial Feminization
0NRM4JZ	ICD-10	Replacement of Right Zygomatic Bone with Synthetic Substitute, Percutaneous Endoscopic Approach	Facial Feminization
0NRM4KZ	ICD-10	Replacement of Right Zygomatic Bone with Nonautologous Tissue Substitute, Percutaneous Endoscopic Approach	Facial Feminization
0NRN07Z	ICD-10	Replacement of Left Zygomatic Bone with Autologous Tissue Substitute, Open Approach	Facial Feminization
0NRN0JZ	ICD-10	Replacement of Left Zygomatic Bone with Synthetic Substitute, Open Approach	Facial Feminization

0NRN0KZ	ICD-10	Replacement of Left Zygomatic Bone with Nonautologous Tissue Substitute, Open Approach	Facial Feminization
0NRN37Z	ICD-10	Replacement of Left Zygomatic Bone with Autologous Tissue Substitute, Percutaneous Approach	Facial Feminization
0NRN3JZ	ICD-10	Replacement of Left Zygomatic Bone with Synthetic Substitute, Percutaneous Approach	Facial Feminization
0NRN3KZ	ICD-10	Replacement of Left Zygomatic Bone with Nonautologous Tissue Substitute, Percutaneous Approach	Facial Feminization
0NRN47Z	ICD-10	Replacement of Left Zygomatic Bone with Autologous Tissue Substitute, Percutaneous Endoscopic Approach	Facial Feminization
0NRN4JZ	ICD-10	Replacement of Left Zygomatic Bone with Synthetic Substitute, Percutaneous Endoscopic Approach	Facial Feminization
0NRN4KZ	ICD-10	Replacement of Left Zygomatic Bone with Nonautologous Tissue Substitute, Percutaneous Endoscopic Approach	Facial Feminization
0NRP0KZ	ICD-10	Replacement of Right Orbit with Nonautologous Tissue Substitute, Open Approach	Facial Feminization
0NRP3KZ	ICD-10	Replacement of Right Orbit with Nonautologous Tissue Substitute, Percutaneous Approach	Facial Feminization
0NRP4KZ	ICD-10	Replacement of Right Orbit with Nonautologous Tissue Substitute, Percutaneous Endoscopic Approach	Facial Feminization
0NRQ0KZ	ICD-10	Replacement of Left Orbit with Nonautologous Tissue Substitute, Open Approach	Facial Feminization
0NRQ3KZ	ICD-10	Replacement of Left Orbit with Nonautologous Tissue Substitute, Percutaneous Approach	Facial Feminization
0NRQ4KZ	ICD-10	Replacement of Left Orbit with Nonautologous Tissue Substitute, Percutaneous Endoscopic Approach	Facial Feminization
0NRR07Z	ICD-10	Replacement of Right Maxilla with Autologous Tissue Substitute, Open Approach	Facial Feminization
0NRR0JZ	ICD-10	Replacement of Right Maxilla with Synthetic Substitute, Open Approach	Facial Feminization
0NRR0KZ	ICD-10	Replacement of Right Maxilla with Nonautologous Tissue Substitute, Open Approach	Facial Feminization
0NRR37Z	ICD-10	Replacement of Right Maxilla with Autologous Tissue Substitute, Percutaneous Approach	Facial Feminization
0NRR3JZ	ICD-10	Replacement of Right Maxilla with Synthetic Substitute, Percutaneous Approach	Facial Feminization
0NRR3KZ	ICD-10	Replacement of Right Maxilla with Nonautologous Tissue Substitute, Percutaneous Approach	Facial Feminization
0NRR47Z	ICD-10	Replacement of Right Maxilla with Autologous Tissue Substitute, Percutaneous Endoscopic Approach	Facial Feminization
0NRR4JZ	ICD-10	Replacement of Right Maxilla with Synthetic Substitute, Percutaneous Endoscopic Approach	Facial Feminization
0NRR4KZ	ICD-10	Replacement of Right Maxilla with Nonautologous Tissue Substitute, Percutaneous Endoscopic Approach	Facial Feminization
0NRS07Z	ICD-10	Replacement of Left Maxilla with Autologous Tissue Substitute, Open Approach	Facial Feminization
0NRS0JZ	ICD-10	Replacement of Left Maxilla with Synthetic Substitute, Open Approach	Facial Feminization
0NRS0KZ	ICD-10	Replacement of Left Maxilla with Nonautologous Tissue Substitute, Open Approach	Facial Feminization
0NRS37Z	ICD-10	Replacement of Left Maxilla with Autologous Tissue Substitute, Percutaneous Approach	Facial Feminization
0NRS3JZ	ICD-10	Replacement of Left Maxilla with Synthetic Substitute, Percutaneous Approach	Facial Feminization
0NRS3KZ	ICD-10	Replacement of Left Maxilla with Nonautologous Tissue Substitute, Percutaneous Approach	Facial Feminization
0NRS47Z	ICD-10	Replacement of Left Maxilla with Autologous Tissue Substitute, Percutaneous Endoscopic Approach	Facial Feminization
0NRS4JZ	ICD-10	Replacement of Left Maxilla with Synthetic Substitute, Percutaneous Endoscopic Approach	Facial Feminization
0NRS4KZ	ICD-10	Replacement of Left Maxilla with Nonautologous Tissue Substitute, Percutaneous Endoscopic Approach	Facial Feminization
0NRX07Z	ICD-10	Replacement of Hyoid Bone with Autologous Tissue Substitute, Open Approach	Facial Feminization
0NRX0JZ	ICD-10	Replacement of Hyoid Bone with Synthetic Substitute, Open Approach	Facial Feminization
0NRX0KZ	ICD-10	Replacement of Hyoid Bone with Nonautologous Tissue Substitute, Open Approach	Facial Feminization
0NRX37Z	ICD-10	Replacement of Hyoid Bone with Autologous Tissue Substitute, Percutaneous Approach	Facial Feminization
0NRX3JZ	ICD-10	Replacement of Hyoid Bone with Synthetic Substitute, Percutaneous Approach	Facial Feminization
0NRX3KZ	ICD-10	Replacement of Hyoid Bone with Nonautologous Tissue Substitute, Percutaneous Approach	Facial Feminization
0NRX47Z	ICD-10	Replacement of Hyoid Bone with Autologous Tissue Substitute, Percutaneous Endoscopic Approach	Facial Feminization
0NRX4JZ	ICD-10	Replacement of Hyoid Bone with Synthetic Substitute, Percutaneous Endoscopic Approach	Facial Feminization
0NRX4KZ	ICD-10	Replacement of Hyoid Bone with Nonautologous Tissue Substitute, Percutaneous Endoscopic Approach	Facial Feminization
0NS00ZZ	ICD-10	Reposition Skull, Open Approach	Facial Feminization
0NS104Z	ICD-10	Reposition Frontal Bone with Internal Fixation Device, Open Approach	Facial Feminization
0NSR04Z	ICD-10	Reposition Maxilla with Internal Fixation Device, Open Approach	Facial Feminization
0NST04Z	ICD-10	Reposition Right Mandible with Internal Fixation Device, Open Approach	Facial Feminization
0NST0ZZ	ICD-10	Reposition Right Mandible, Open Approach	Facial Feminization
0NSV04Z	ICD-10	Reposition Left Mandible with Internal Fixation Device, Open Approach	Facial Feminization

0NSV0ZZ	ICD-10	Reposition Left Mandible, Open Approach	Facial Feminization
0NU10JZ	ICD-10	Supplement Frontal Bone with Synthetic Substitute, Open Approach	Facial Feminization
0NUC07Z	ICD-10	Supplement Right Sphenoid Bone with Autologous Tissue Substitute, Open Approach	Facial Feminization
0NUC0JZ	ICD-10	Supplement Right Sphenoid Bone with Synthetic Substitute, Open Approach	Facial Feminization
0NUC0KZ	ICD-10	Supplement Right Sphenoid Bone with Nonautologous Tissue Substitute, Open Approach	Facial Feminization
0NUC37Z	ICD-10	Supplement Right Sphenoid Bone with Autologous Tissue Substitute, Percutaneous Approach	Facial Feminization
0NUC3JZ	ICD-10	Supplement Right Sphenoid Bone with Synthetic Substitute, Percutaneous Approach	Facial Feminization
0NUC3KZ	ICD-10	Supplement Right Sphenoid Bone with Nonautologous Tissue Substitute, Percutaneous Approach	Facial Feminization
0NUC47Z	ICD-10	Supplement Right Sphenoid Bone with Autologous Tissue Substitute, Percutaneous Endoscopic Approach	Facial Feminization
0NUC4JZ	ICD-10	Supplement Right Sphenoid Bone with Synthetic Substitute, Percutaneous Endoscopic Approach	Facial Feminization
0NUC4KZ	ICD-10	Supplement Right Sphenoid Bone with Nonautologous Tissue Substitute, Percutaneous Endoscopic Approach	Facial Feminization
0NUD07Z	ICD-10	Supplement Left Sphenoid Bone with Autologous Tissue Substitute, Open Approach	Facial Feminization
0NUD0JZ	ICD-10	Supplement Left Sphenoid Bone with Synthetic Substitute, Open Approach	Facial Feminization
0NUD0KZ	ICD-10	Supplement Left Sphenoid Bone with Nonautologous Tissue Substitute, Open Approach	Facial Feminization
0NUD37Z	ICD-10	Supplement Left Sphenoid Bone with Autologous Tissue Substitute, Percutaneous Approach	Facial Feminization
0NUD3JZ	ICD-10	Supplement Left Sphenoid Bone with Synthetic Substitute, Percutaneous Approach	Facial Feminization
0NUD3KZ	ICD-10	Supplement Left Sphenoid Bone with Nonautologous Tissue Substitute, Percutaneous Approach	Facial Feminization
0NUD47Z	ICD-10	Supplement Left Sphenoid Bone with Autologous Tissue Substitute, Percutaneous Endoscopic Approach	Facial Feminization
0NUD4JZ	ICD-10	Supplement Left Sphenoid Bone with Synthetic Substitute, Percutaneous Endoscopic Approach	Facial Feminization
0NUD4KZ	ICD-10	Supplement Left Sphenoid Bone with Nonautologous Tissue Substitute, Percutaneous Endoscopic Approach	Facial Feminization
0NUF07Z	ICD-10	Supplement Right Ethmoid Bone with Autologous Tissue Substitute, Open Approach	Facial Feminization
0NUF0JZ	ICD-10	Supplement Right Ethmoid Bone with Synthetic Substitute, Open Approach	Facial Feminization
0NUF0KZ	ICD-10	Supplement Right Ethmoid Bone with Nonautologous Tissue Substitute, Open Approach	Facial Feminization
0NUF37Z	ICD-10	Supplement Right Ethmoid Bone with Autologous Tissue Substitute, Percutaneous Approach	Facial Feminization
0NUF3JZ	ICD-10	Supplement Right Ethmoid Bone with Synthetic Substitute, Percutaneous Approach	Facial Feminization
0NUF3KZ	ICD-10	Supplement Right Ethmoid Bone with Nonautologous Tissue Substitute, Percutaneous Approach	Facial Feminization
0NUF47Z	ICD-10	Supplement Right Ethmoid Bone with Autologous Tissue Substitute, Percutaneous Endoscopic Approach	Facial Feminization
0NUF4JZ	ICD-10	Supplement Right Ethmoid Bone with Synthetic Substitute, Percutaneous Endoscopic Approach	Facial Feminization
0NUF4KZ	ICD-10	Supplement Right Ethmoid Bone with Nonautologous Tissue Substitute, Percutaneous Endoscopic Approach	Facial Feminization
0NUG07Z	ICD-10	Supplement Left Ethmoid Bone with Autologous Tissue Substitute, Open Approach	Facial Feminization
0NUG0JZ	ICD-10	Supplement Left Ethmoid Bone with Synthetic Substitute, Open Approach	Facial Feminization
0NUG0KZ	ICD-10	Supplement Left Ethmoid Bone with Nonautologous Tissue Substitute, Open Approach	Facial Feminization
0NUG37Z	ICD-10	Supplement Left Ethmoid Bone with Autologous Tissue Substitute, Percutaneous Approach	Facial Feminization
0NUG3JZ	ICD-10	Supplement Left Ethmoid Bone with Synthetic Substitute, Percutaneous Approach	Facial Feminization
0NUG3KZ	ICD-10	Supplement Left Ethmoid Bone with Nonautologous Tissue Substitute, Percutaneous Approach	Facial Feminization
0NUG47Z	ICD-10	Supplement Left Ethmoid Bone with Autologous Tissue Substitute, Percutaneous Endoscopic Approach	Facial Feminization
0NUG4JZ	ICD-10	Supplement Left Ethmoid Bone with Synthetic Substitute, Percutaneous Endoscopic Approach	Facial Feminization
0NUG4KZ	ICD-10	Supplement Left Ethmoid Bone with Nonautologous Tissue Substitute, Percutaneous Endoscopic Approach	Facial Feminization
0NUH07Z	ICD-10	Supplement Right Lacrimal Bone with Autologous Tissue Substitute, Open Approach	Facial Feminization
0NUH0JZ	ICD-10	Supplement Right Lacrimal Bone with Synthetic Substitute, Open Approach	Facial Feminization
0NUH0KZ	ICD-10	Supplement Right Lacrimal Bone with Nonautologous Tissue Substitute, Open Approach	Facial Feminization
0NUH37Z	ICD-10	Supplement Right Lacrimal Bone with Autologous Tissue Substitute, Percutaneous Approach	Facial Feminization
0NUH3JZ	ICD-10	Supplement Right Lacrimal Bone with Synthetic Substitute, Percutaneous Approach	Facial Feminization
0NUH3KZ	ICD-10	Supplement Right Lacrimal Bone with Nonautologous Tissue Substitute, Percutaneous Approach	Facial Feminization
0NUH47Z	ICD-10	Supplement Right Lacrimal Bone with Autologous Tissue Substitute, Percutaneous Endoscopic Approach	Facial Feminization
0NUH4JZ	ICD-10	Supplement Right Lacrimal Bone with Synthetic Substitute, Percutaneous Endoscopic Approach	Facial Feminization

0NUH4KZ	ICD-10	Supplement Right Lacrimal Bone with Nonautologous Tissue Substitute, Percutaneous Endoscopic Approach	Facial Feminization
0NUJ07Z	ICD-10	Supplement Left Lacrimal Bone with Autologous Tissue Substitute, Open Approach	Facial Feminization
0NUJ0JZ	ICD-10	Supplement Left Lacrimal Bone with Synthetic Substitute, Open Approach	Facial Feminization
0NUJ0KZ	ICD-10	Supplement Left Lacrimal Bone with Nonautologous Tissue Substitute, Open Approach	Facial Feminization
0NUJ37Z	ICD-10	Supplement Left Lacrimal Bone with Autologous Tissue Substitute, Percutaneous Approach	Facial Feminization
0NUJ3JZ	ICD-10	Supplement Left Lacrimal Bone with Synthetic Substitute, Percutaneous Approach	Facial Feminization
0NUJ3KZ	ICD-10	Supplement Left Lacrimal Bone with Nonautologous Tissue Substitute, Percutaneous Approach	Facial Feminization
0NUJ47Z	ICD-10	Supplement Left Lacrimal Bone with Autologous Tissue Substitute, Percutaneous Endoscopic Approach	Facial Feminization
0NUJ4JZ	ICD-10	Supplement Left Lacrimal Bone with Synthetic Substitute, Percutaneous Endoscopic Approach	Facial Feminization
0NUJ4KZ	ICD-10	Supplement Left Lacrimal Bone with Nonautologous Tissue Substitute, Percutaneous Endoscopic Approach	Facial Feminization
0NUK07Z	ICD-10	Supplement Right Palatine Bone with Autologous Tissue Substitute, Open Approach	Facial Feminization
0NUK0JZ	ICD-10	Supplement Right Palatine Bone with Synthetic Substitute, Open Approach	Facial Feminization
0NUK0KZ	ICD-10	Supplement Right Palatine Bone with Nonautologous Tissue Substitute, Open Approach	Facial Feminization
0NUK37Z	ICD-10	Supplement Right Palatine Bone with Autologous Tissue Substitute, Percutaneous Approach	Facial Feminization
0NUK3JZ	ICD-10	Supplement Right Palatine Bone with Synthetic Substitute, Percutaneous Approach	Facial Feminization
0NUK3KZ	ICD-10	Supplement Right Palatine Bone with Nonautologous Tissue Substitute, Percutaneous Approach	Facial Feminization
0NUK47Z	ICD-10	Supplement Right Palatine Bone with Autologous Tissue Substitute, Percutaneous Endoscopic Approach	Facial Feminization
0NUK4JZ	ICD-10	Supplement Right Palatine Bone with Synthetic Substitute, Percutaneous Endoscopic Approach	Facial Feminization
0NUK4KZ	ICD-10	Supplement Right Palatine Bone with Nonautologous Tissue Substitute, Percutaneous Endoscopic Approach	Facial Feminization
0NUL07Z	ICD-10	Supplement Left Palatine Bone with Autologous Tissue Substitute, Open Approach	Facial Feminization
0NUL0JZ	ICD-10	Supplement Left Palatine Bone with Synthetic Substitute, Open Approach	Facial Feminization
0NUL0KZ	ICD-10	Supplement Left Palatine Bone with Nonautologous Tissue Substitute, Open Approach	Facial Feminization
0NUL37Z	ICD-10	Supplement Left Palatine Bone with Autologous Tissue Substitute, Percutaneous Approach	Facial Feminization
0NUL3JZ	ICD-10	Supplement Left Palatine Bone with Synthetic Substitute, Percutaneous Approach	Facial Feminization
0NUL3KZ	ICD-10	Supplement Left Palatine Bone with Nonautologous Tissue Substitute, Percutaneous Approach	Facial Feminization
0NUL47Z	ICD-10	Supplement Left Palatine Bone with Autologous Tissue Substitute, Percutaneous Endoscopic Approach	Facial Feminization
0NUL4JZ	ICD-10	Supplement Left Palatine Bone with Synthetic Substitute, Percutaneous Endoscopic Approach	Facial Feminization
0NUL4KZ	ICD-10	Supplement Left Palatine Bone with Nonautologous Tissue Substitute, Percutaneous Endoscopic Approach	Facial Feminization
0NUM07Z	ICD-10	Supplement Right Zygomatic Bone with Autologous Tissue Substitute, Open Approach	Facial Feminization
0NUM0JZ	ICD-10	Supplement Right Zygomatic Bone with Synthetic Substitute, Open Approach	Facial Feminization
0NUM0KZ	ICD-10	Supplement Right Zygomatic Bone with Nonautologous Tissue Substitute, Open Approach	Facial Feminization
0NUM37Z	ICD-10	Supplement Right Zygomatic Bone with Autologous Tissue Substitute, Percutaneous Approach	Facial Feminization
0NUM3JZ	ICD-10	Supplement Right Zygomatic Bone with Synthetic Substitute, Percutaneous Approach	Facial Feminization
0NUM3KZ	ICD-10	Supplement Right Zygomatic Bone with Nonautologous Tissue Substitute, Percutaneous Approach	Facial Feminization
0NUM47Z	ICD-10	Supplement Right Zygomatic Bone with Autologous Tissue Substitute, Percutaneous Endoscopic Approach	Facial Feminization
0NUM4JZ	ICD-10	Supplement Right Zygomatic Bone with Synthetic Substitute, Percutaneous Endoscopic Approach	Facial Feminization
0NUM4KZ	ICD-10	Supplement Right Zygomatic Bone with Nonautologous Tissue Substitute, Percutaneous Endoscopic Approach	Facial Feminization
0NUN07Z	ICD-10	Supplement Left Zygomatic Bone with Autologous Tissue Substitute, Open Approach	Facial Feminization
0NUN0JZ	ICD-10	Supplement Left Zygomatic Bone with Synthetic Substitute, Open Approach	Facial Feminization
0NUN0KZ	ICD-10	Supplement Left Zygomatic Bone with Nonautologous Tissue Substitute, Open Approach	Facial Feminization
0NUN37Z	ICD-10	Supplement Left Zygomatic Bone with Autologous Tissue Substitute, Percutaneous Approach	Facial Feminization
0NUN3JZ	ICD-10	Supplement Left Zygomatic Bone with Synthetic Substitute, Percutaneous Approach	Facial Feminization
0NUN3KZ	ICD-10	Supplement Left Zygomatic Bone with Nonautologous Tissue Substitute, Percutaneous Approach	Facial Feminization
0NUN47Z	ICD-10	Supplement Left Zygomatic Bone with Autologous Tissue Substitute, Percutaneous Endoscopic Approach	Facial Feminization
0NUN4JZ	ICD-10	Supplement Left Zygomatic Bone with Synthetic Substitute, Percutaneous Endoscopic Approach	Facial Feminization
0NUN4KZ	ICD-10	Supplement Left Zygomatic Bone with Nonautologous Tissue Substitute, Percutaneous Endoscopic Approach	Facial Feminization

0NUP07Z	ICD-10	Supplement Right Orbit with Autologous Tissue Substitute, Open Approach	Facial Feminization
0NUP0KZ	ICD-10	Supplement Right Orbit with Nonautologous Tissue Substitute, Open Approach	Facial Feminization
0NUP37Z	ICD-10	Supplement Right Orbit with Autologous Tissue Substitute, Percutaneous Approach	Facial Feminization
0NUP3KZ	ICD-10	Supplement Right Orbit with Nonautologous Tissue Substitute, Percutaneous Approach	Facial Feminization
0NUP47Z	ICD-10	Supplement Right Orbit with Autologous Tissue Substitute, Percutaneous Endoscopic Approach	Facial Feminization
0NUP4KZ	ICD-10	Supplement Right Orbit with Nonautologous Tissue Substitute, Percutaneous Endoscopic Approach	Facial Feminization
0NUQ07Z	ICD-10	Supplement Left Orbit with Autologous Tissue Substitute, Open Approach	Facial Feminization
0NUQ0KZ	ICD-10	Supplement Left Orbit with Nonautologous Tissue Substitute, Open Approach	Facial Feminization
0NUQ37Z	ICD-10	Supplement Left Orbit with Autologous Tissue Substitute, Percutaneous Approach	Facial Feminization
0NUQ3KZ	ICD-10	Supplement Left Orbit with Nonautologous Tissue Substitute, Percutaneous Approach	Facial Feminization
0NUQ47Z	ICD-10	Supplement Left Orbit with Autologous Tissue Substitute, Percutaneous Endoscopic Approach	Facial Feminization
0NUQ4KZ	ICD-10	Supplement Left Orbit with Nonautologous Tissue Substitute, Percutaneous Endoscopic Approach	Facial Feminization
0NUR07Z	ICD-10	Supplement Right Maxilla with Autologous Tissue Substitute, Open Approach	Facial Feminization
0NUR0JZ	ICD-10	Supplement Right Maxilla with Synthetic Substitute, Open Approach	Facial Feminization
0NUR0KZ	ICD-10	Supplement Right Maxilla with Nonautologous Tissue Substitute, Open Approach	Facial Feminization
0NUR37Z	ICD-10	Supplement Right Maxilla with Autologous Tissue Substitute, Percutaneous Approach	Facial Feminization
0NUR3JZ	ICD-10	Supplement Right Maxilla with Synthetic Substitute, Percutaneous Approach	Facial Feminization
0NUR3KZ	ICD-10	Supplement Right Maxilla with Nonautologous Tissue Substitute, Percutaneous Approach	Facial Feminization
0NUR47Z	ICD-10	Supplement Right Maxilla with Autologous Tissue Substitute, Percutaneous Endoscopic Approach	Facial Feminization
0NUR4JZ	ICD-10	Supplement Right Maxilla with Synthetic Substitute, Percutaneous Endoscopic Approach	Facial Feminization
0NUR4KZ	ICD-10	Supplement Right Maxilla with Nonautologous Tissue Substitute, Percutaneous Endoscopic Approach	Facial Feminization
0NUS07Z	ICD-10	Supplement Left Maxilla with Autologous Tissue Substitute, Open Approach	Facial Feminization
0NUS0JZ	ICD-10	Supplement Left Maxilla with Synthetic Substitute, Open Approach	Facial Feminization
0NUS0KZ	ICD-10	Supplement Left Maxilla with Nonautologous Tissue Substitute, Open Approach	Facial Feminization
0NUS37Z	ICD-10	Supplement Left Maxilla with Autologous Tissue Substitute, Percutaneous Approach	Facial Feminization
0NUS3JZ	ICD-10	Supplement Left Maxilla with Synthetic Substitute, Percutaneous Approach	Facial Feminization
0NUS3KZ	ICD-10	Supplement Left Maxilla with Nonautologous Tissue Substitute, Percutaneous Approach	Facial Feminization
0NUS47Z	ICD-10	Supplement Left Maxilla with Autologous Tissue Substitute, Percutaneous Endoscopic Approach	Facial Feminization
0NUS4JZ	ICD-10	Supplement Left Maxilla with Synthetic Substitute, Percutaneous Endoscopic Approach	Facial Feminization
0NUS4KZ	ICD-10	Supplement Left Maxilla with Nonautologous Tissue Substitute, Percutaneous Endoscopic Approach	Facial Feminization
0NUX07Z	ICD-10	Supplement Hyoid Bone with Autologous Tissue Substitute, Open Approach	Facial Feminization
0NUX0JZ	ICD-10	Supplement Hyoid Bone with Synthetic Substitute, Open Approach	Facial Feminization
0NUX0KZ	ICD-10	Supplement Hyoid Bone with Nonautologous Tissue Substitute, Open Approach	Facial Feminization
0NUX37Z	ICD-10	Supplement Hyoid Bone with Autologous Tissue Substitute, Percutaneous Approach	Facial Feminization
0NUX3JZ	ICD-10	Supplement Hyoid Bone with Synthetic Substitute, Percutaneous Approach	Facial Feminization
0NUX3KZ	ICD-10	Supplement Hyoid Bone with Nonautologous Tissue Substitute, Percutaneous Approach	Facial Feminization
0NUX47Z	ICD-10	Supplement Hyoid Bone with Autologous Tissue Substitute, Percutaneous Endoscopic Approach	Facial Feminization
0NUX4JZ	ICD-10	Supplement Hyoid Bone with Synthetic Substitute, Percutaneous Endoscopic Approach	Facial Feminization
0NUX4KZ	ICD-10	Supplement Hyoid Bone with Nonautologous Tissue Substitute, Percutaneous Endoscopic Approach	Facial Feminization
0RNC0ZZ	ICD-10	Release Right Temporomandibular Joint, Open Approach	Facial Feminization
0RNC3ZZ	ICD-10	Release Right Temporomandibular Joint, Percutaneous Approach	Facial Feminization
0RNC4ZZ	ICD-10	Release Right Temporomandibular Joint, Percutaneous Endoscopic Approach	Facial Feminization
0RND0ZZ	ICD-10	Release Left Temporomandibular Joint, Open Approach	Facial Feminization
0RND3ZZ	ICD-10	Release Left Temporomandibular Joint, Percutaneous Approach	Facial Feminization
0RND4ZZ	ICD-10	Release Left Temporomandibular Joint, Percutaneous Endoscopic Approach	Facial Feminization
0W0007Z	ICD-10	Alteration of Head with Autologous Tissue Substitute, Open Approach	Facial Feminization

0W020ZZ	ICD-10	Alteration of Face, Open Approach	Facial Feminization
0W0407Z	ICD-10	Alteration of Upper Jaw with Autologous Tissue Substitute, Open Approach	Facial Feminization
0W040JZ	ICD-10	Alteration of Upper Jaw with Synthetic Substitute, Open Approach	Facial Feminization
0W040KZ	ICD-10	Alteration of Upper Jaw with Nonautologous Tissue Substitute, Open Approach	Facial Feminization
0W040ZZ	ICD-10	Alteration of Upper Jaw, Open Approach	Facial Feminization
0W0437Z	ICD-10	Alteration of Upper Jaw with Autologous Tissue Substitute, Percutaneous Approach	Facial Feminization
0W043JZ	ICD-10	Alteration of Upper Jaw with Synthetic Substitute, Percutaneous Approach	Facial Feminization
0W043KZ	ICD-10	Alteration of Upper Jaw with Nonautologous Tissue Substitute, Percutaneous Approach	Facial Feminization
0W043ZZ	ICD-10	Alteration of Upper Jaw, Percutaneous Approach	Facial Feminization
0W0447Z	ICD-10	Alteration of Upper Jaw with Autologous Tissue Substitute, Percutaneous Endoscopic Approach	Facial Feminization
0W044JZ	ICD-10	Alteration of Upper Jaw with Synthetic Substitute, Percutaneous Endoscopic Approach	Facial Feminization
0W044KZ	ICD-10	Alteration of Upper Jaw with Nonautologous Tissue Substitute, Percutaneous Endoscopic Approach	Facial Feminization
0W044ZZ	ICD-10	Alteration of Upper Jaw, Percutaneous Endoscopic Approach	Facial Feminization
0W0507Z	ICD-10	Alteration of Lower Jaw with Autologous Tissue Substitute, Open Approach	Facial Feminization
0W050JZ	ICD-10	Alteration of Lower Jaw with Synthetic Substitute, Open Approach	Facial Feminization
0W050KZ	ICD-10	Alteration of Lower Jaw with Nonautologous Tissue Substitute, Open Approach	Facial Feminization
0W050ZZ	ICD-10	Alteration of Lower Jaw, Open Approach	Facial Feminization
0W0537Z	ICD-10	Alteration of Lower Jaw with Autologous Tissue Substitute, Percutaneous Approach	Facial Feminization
0W053JZ	ICD-10	Alteration of Lower Jaw with Synthetic Substitute, Percutaneous Approach	Facial Feminization
0W053KZ	ICD-10	Alteration of Lower Jaw with Nonautologous Tissue Substitute, Percutaneous Approach	Facial Feminization
0W053ZZ	ICD-10	Alteration of Lower Jaw, Percutaneous Approach	Facial Feminization
0W0547Z	ICD-10	Alteration of Lower Jaw with Autologous Tissue Substitute, Percutaneous Endoscopic Approach	Facial Feminization
0W054JZ	ICD-10	Alteration of Lower Jaw with Synthetic Substitute, Percutaneous Endoscopic Approach	Facial Feminization
0W054KZ	ICD-10	Alteration of Lower Jaw with Nonautologous Tissue Substitute, Percutaneous Endoscopic Approach	Facial Feminization
0W054ZZ	ICD-10	Alteration of Lower Jaw, Percutaneous Endoscopic Approach	Facial Feminization
0W060ZZ	ICD-10	Alteration of Neck, Open Approach	Facial Feminization
0W064ZZ	ICD-10	Alteration of Neck, Percutaneous Endoscopic Approach	Facial Feminization
0WU407Z	ICD-10	Supplement Upper Jaw with Autologous Tissue Substitute, Open Approach	Facial Feminization
0WU40JZ	ICD-10	Supplement Upper Jaw with Synthetic Substitute, Open Approach	Facial Feminization
0WU40KZ	ICD-10	Supplement Upper Jaw with Nonautologous Tissue Substitute, Open Approach	Facial Feminization
0WU447Z	ICD-10	Supplement Upper Jaw with Autologous Tissue Substitute, Percutaneous Endoscopic Approach	Facial Feminization
0WU44JZ	ICD-10	Supplement Upper Jaw with Synthetic Substitute, Percutaneous Endoscopic Approach	Facial Feminization
0WU44KZ	ICD-10	Supplement Upper Jaw with Nonautologous Tissue Substitute, Percutaneous Endoscopic Approach	Facial Feminization
0WU507Z	ICD-10	Supplement Lower Jaw with Autologous Tissue Substitute, Open Approach	Facial Feminization
0WU50JZ	ICD-10	Supplement Lower Jaw with Synthetic Substitute, Open Approach	Facial Feminization
0WU50KZ	ICD-10	Supplement Lower Jaw with Nonautologous Tissue Substitute, Open Approach	Facial Feminization
0WU547Z	ICD-10	Supplement Lower Jaw with Autologous Tissue Substitute, Percutaneous Endoscopic Approach	Facial Feminization
0WU54JZ	ICD-10	Supplement Lower Jaw with Synthetic Substitute, Percutaneous Endoscopic Approach	Facial Feminization
0WU54KZ	ICD-10	Supplement Lower Jaw with Nonautologous Tissue Substitute, Percutaneous Endoscopic Approach	Facial Feminization
8E09XBG	ICD-10	Computer Assisted Procedure of Head and Neck Region, With Computerized Tomography	Facial Feminization
15776	CPT	Hair transplant >15 punch grafts	Facial Feminization
15819	CPT	Plastic surgery neck	Facial Feminization
15820	CPT	Blepharoplasty, lower eyelid	Facial Feminization
15821	CPT	Blepharoplasty, lower eyelid; with extensive herniated fat pad	Facial Feminization
15822	CPT	Blepharoplasty, upper eyelid	Facial Feminization
15823	CPT	Blepharoplasty, upper eyelid; with excessive skin weighting down lid	Facial Feminization

15824	CPT	Rhytidectomy; forehead	Facial Feminization
15825	CPT	Rhytidectomy; neck with platysmal tightening (platysmal flap, P-flap)	Facial Feminization
15826	CPT	Rhytidectomy; glabellar frown lines	Facial Feminization
15828	CPT	Rhytidectomy; cheek, chin, and neck	Facial Feminization
15876	CPT	Suction assisted lipectomy; head and neck	Facial Feminization
21025	CPT	Excision of bone lower jaw	Facial Feminization
21026	CPT	Excision of facial bones	Facial Feminization
21083	CPT	Impression and custom preparation; palatal lift prosthesis	Facial Feminization
21087	CPT	Impression and custom preparation; nasal prosthesis	Facial Feminization
21089	CPT	Prepare face/oral prosthesis	Facial Feminization
21120	CPT	Genioplasty; augmentation (autograft, allograft, prosthetic material)	Facial Feminization
21121	CPT	Genioplasty; sliding osteotomy, single piece	Facial Feminization
21122	CPT	Genioplasty; sliding osteotomies, 2 or more osteotomies (eg, wedge excision or bone wedge reversal for asymmetrical chin)	Facial Feminization
21123	CPT	Genioplasty; sliding, augmentation with interpositional bone grafts (includes obtaining autografts)	Facial Feminization
21125	CPT	Augmentation, mandibular body or angle; prosthetic material	Facial Feminization
21127	CPT	Augmentation, mandibular body or angle; with bone graft, onlay or interpositional (includes obtaining autograft)	Facial Feminization
21137	CPT	Reduction forehead; contouring only	Facial Feminization
21138	CPT	Reduction forehead; contouring and application of prosthetic material or bone graft (includes obtaining autograft)	Facial Feminization
21139	CPT	Reduction forehead; contouring and setback of anterior frontal sinus wall	Facial Feminization
21141	CPT	Reconstruction midface, LeFort I; single piece, segment movement in any direction (eg, for Long Face Syndrome), without bor	Facial Feminization
21142	CPT	Reconstruction midface, LeFort I; 2 pieces, segment movement in any direction, without bone graft	Facial Feminization
21143	CPT	Reconstruction midface, LeFort I; 3 or more pieces, segment movement in any direction, without bone graft	Facial Feminization
21145	CPT	Reconstruction midface, LeFort I; single piece, segment movement in any direction, requiring bone grafts (includes obtaining	Facial Feminization
21146	CPT	Reconstruction midface, LeFort I; 2 pieces, segment movement in any direction, requiring bone grafts (includes obtaining aut	Facial Feminization
21147	CPT	Reconstruction midface, LeFort I; 3 or more pieces, segment movement in any direction, requiring bone grafts (includes obtai	Facial Feminization
21150	CPT	Reconstruction midface, LeFort II; anterior intrusion (eg, Treacher-Collins Syndrome)	Facial Feminization
21151	CPT	Reconstruction midface, LeFort II; any direction, requiring bone grafts (includes obtaining autografts)	Facial Feminization
21154	CPT	Reconstruction midface, LeFort III (extracranial), any type, requiring bone grafts (includes obtaining autografts); without LeFo	Facial Feminization
21155	CPT	Reconstruction midface, LeFort III (extracranial), any type, requiring bone grafts (includes obtaining autografts); with LeFort I	Facial Feminization
21159	CPT	Reconstruction midface, LeFort III (extra and intracranial) with forehead advancement (eg, mono bloc), requiring bone grafts	Facial Feminization
21160	CPT	Reconstruction midface, LeFort III (extra and intracranial) with forehead advancement (eg, mono bloc), requiring bone grafts	Facial Feminization
21172	CPT	Reconstruction superior-lateral orbital rim and lower forehead, advancement or alteration, with or without grafts (includes ol	Facial Feminization
21175	CPT	Reconstruction, bifrontal, superior-lateral orbital rims and lower forehead, advancement or alteration (eg, plagiocephaly, trig	Facial Feminization
21179	CPT	Reconstruction, entire or majority of forehead and/or supraorbital rims; with grafts (allograft or prosthetic material)	Facial Feminization
21180	CPT	Reconstruction, entire or majority of forehead and/or supraorbital rims; with autograft (includes obtaining grafts)	Facial Feminization
21188	CPT	Reconstruction midface, osteotomies (other than LeFort type) and bone grafts (includes obtaining autografts)	Facial Feminization
21193	CPT	Reconstruction of lower jaw without graft	Facial Feminization
21194	CPT	Reconstruction of lower jaw with graft	Facial Feminization
21195	CPT	Reconstruction of lower jaw without fixation	Facial Feminization
21196	CPT	Reconstruction of lower jaw with fixation	Facial Feminization
21198	CPT	Reconstruction of lower jaw segment	Facial Feminization
21208	CPT	Osteoplasty, facial bones; augmentation (autograft, allograft, or prosthetic implant)	Facial Feminization
21209	CPT	Osteoplasty, facial bones; reduction	Facial Feminization
21210	CPT	Graft, bone; nasal, maxillary or malar areas (includes obtaining graft)	Facial Feminization
21230	CPT	Graft; rib cartilage, autogenous, to face, chin, nose or ear (includes obtaining graft)	Facial Feminization
21235	CPT	Graft; ear cartilage, autogenous, to nose or ear (includes obtaining graft)	Facial Feminization

21244	CPT	Reconstruction of mandible, extraoral, with transosteal bone plate (eg, mandibular staple bone plate)	Facial Feminization
21245	CPT	Reconstruction of mandible or maxilla, subperiosteal implant; partial	Facial Feminization
21246	CPT	Reconstruction of mandible or maxilla, subperiosteal implant; complete	Facial Feminization
21248	CPT	Reconstruction of mandible or maxilla, endosteal implant (eg, blade, cylinder); partial	Facial Feminization
21249	CPT	Reconstruction of mandible or maxilla, endosteal implant (eg, blade, cylinder); complete	Facial Feminization
21270	CPT	Malar augmentation, prosthetic material	Facial Feminization
21295	CPT	Revision of jaw muscle/bone	Facial Feminization
21296	CPT	Revision of jaw muscle/bone	Facial Feminization
21299	CPT	Cranio/maxillofacial surgery	Facial Feminization
21335	CPT	Open treatment of nasal fracture	Facial Feminization
21499	CPT	Head surgery procedure	Facial Feminization
30130	CPT	Excise inferior turbinate	Facial Feminization
30140	CPT	Resect inferior turbinate	Facial Feminization
30400	CPT	Rhinoplasty, primary; lateral and alar cartilages and/or elevation of nasal tip	Facial Feminization
30410	CPT	Rhinoplasty, primary; complete, external parts including bony pyramid, lateral and alar cartilages, and/or elevation of nasal ti	Facial Feminization
30420	CPT	Rhinoplasty, primary; including major septal repair	Facial Feminization
30430	CPT	Rhinoplasty, secondary; minor revision (small amount of nasal tip work)	Facial Feminization
30435	CPT	Rhinoplasty, secondary; intermediate revision (bony work with osteotomies)	Facial Feminization
30450	CPT	Rhinoplasty, secondary; major revision (nasal tip work and osteotomies)	Facial Feminization
30462	CPT	Revision of nose	Facial Feminization
30520	CPT	Repair of nasal septum	Facial Feminization
30930	CPT	Therapeutic fixation of nasal inferior turbinate	Facial Feminization
30999	CPT	Nasal surgery procedure	Facial Feminization
31599	CPT	Unlisted procedure, larynx	Facial Feminization
31899	CPT	Airways surgical procedure	Facial Feminization
40500	CPT	Partial excision of lip	Facial Feminization
40510	CPT	Partial excision of lip	Facial Feminization
40650	CPT	Repair lip	Facial Feminization
40652	CPT	Repair lip	Facial Feminization
40799	CPT	Lip surgery procedure	Facial Feminization
67900	CPT	Repair of brow ptosis (supraciliary, mid-forehead or coronal approach)	Facial Feminization
67901	CPT	Repair of blepharoptosis; frontalis muscle technique with suture or other material (eg, banked fascia)	Facial Feminization
67902	CPT	Repair of blepharoptosis; frontalis muscle technique with autologous fascial sling (includes obtaining fascia)	Facial Feminization
67903	CPT	Repair of blepharoptosis; (tarso) levator resection or advancement, internal approach	Facial Feminization
67904	CPT	Repair of blepharoptosis; (tarso) levator resection or advancement, external approach	Facial Feminization
67906	CPT	Repair of blepharoptosis; superior rectus technique with fascial sling (includes obtaining fascia)	Facial Feminization
67908	CPT	Repair of blepharoptosis; conjunctivo-tarso-Muller's muscle-levator resection (eg, Fasanella-Servat type)	Facial Feminization
67999	CPT	Revision of eyelid	Facial Feminization
69300	CPT	Otoplasty, protruding ear, with or without size reduction	Facial Feminization
6561	ICD-9	Other removal of both ovaries and tubes at same operative episode	Hysterectomy
6563	ICD-9	Laparoscopic removal of both ovaries and tubes at same operative episode	Hysterectomy
6651	ICD-9	Removal of both fallopian tubes at same operative episode	Hysterectomy
6831	ICD-9	Laparoscopic supracervical hysterectomy	Hysterectomy
6839	ICD-9	Other and unspecified subtotal abdominal hysterectomy	Hysterectomy
6841	ICD-9	Laparoscopic total abdominal hysterectomy	Hysterectomy
6849	ICD-9	Other and unspecified total abdominal hysterectomy	Hysterectomy

6851	ICD-9	Laparoscopically assisted vaginal hysterectomy	Hysterectomy
6859	ICD-9	Other and unspecified vaginal hysterectomy	Hysterectomy
6861	ICD-9	Laparoscopic radical abdominal hysterectomy	Hysterectomy
6869	ICD-9	Other and unspecified radical abdominal hysterectomy	Hysterectomy
6871	ICD-9	Laparoscopic radical vaginal hysterectomy	Hysterectomy
6879	ICD-9	Other and unspecified radical vaginal hysterectomy	Hysterectomy
0UB14ZZ	ICD-10	Excision of Left Ovary, Percutaneous Endoscopic Approach	Hysterectomy
0UB20ZZ	ICD-10	Excision of Bilateral Ovaries, Open Approach	Hysterectomy
0UB24ZZ	ICD-10	Excision of Bilateral Ovaries, Percutaneous Endoscopic Approach	Hysterectomy
0UB70ZZ	ICD-10	Excision of Bilateral Fallopian Tubes, Open Approach	Hysterectomy
0UB74ZZ	ICD-10	Excision of Bilateral Fallopian Tubes, Percutaneous Endoscopic Approach	Hysterectomy
0UT0FZZ	ICD-10	Resection of Right Ovary, Via Natural or Artificial Opening With Percutaneous Endoscopic Assistance	Hysterectomy
0UT20ZZ	ICD-10	Resection of Bilateral Ovaries, Open Approach	Hysterectomy
0UT24ZZ	ICD-10	Resection of Bilateral Ovaries, Percutaneous Endoscopic Approach	Hysterectomy
0UT27ZZ	ICD-10	Resection of Bilateral Ovaries, Via Natural or Artificial Opening	Hysterectomy
0UT28ZZ	ICD-10	Resection of Bilateral Ovaries, Via Natural or Artificial Opening Endoscopic	Hysterectomy
0UT2FZZ	ICD-10	Resection of Bilateral Ovaries, Via Natural or Artificial Opening With Percutaneous Endoscopic Assistance	Hysterectomy
0UT6FZZ	ICD-10	Resection of Left Fallopian Tube, Via Natural or Artificial Opening With Percutaneous Endoscopic Assistance	Hysterectomy
0UT70ZZ	ICD-10	Resection of Bilateral Fallopian Tubes, Open Approach	Hysterectomy
0UT74ZZ	ICD-10	Resection of Bilateral Fallopian Tubes, Percutaneous Endoscopic Approach	Hysterectomy
0UT77ZZ	ICD-10	Resection of Bilateral Fallopian Tubes, Via Natural or Artificial Opening	Hysterectomy
0UT78ZZ	ICD-10	Resection of Bilateral Fallopian Tubes, Via Natural or Artificial Opening Endoscopic	Hysterectomy
0UT7FZZ	ICD-10	Resection of Bilateral Fallopian Tubes, Via Natural or Artificial Opening With Percutaneous Endoscopic Assistance	Hysterectomy
0UT90ZZ	ICD-10	Resection of Uterus, Open Approach	Hysterectomy
0UT94ZZ	ICD-10	Resection of Uterus, Percutaneous Endoscopic Approach	Hysterectomy
0UT97ZZ	ICD-10	Resection of Uterus, Via Natural or Artificial Opening	Hysterectomy
0UT98ZZ	ICD-10	Resection of Uterus, Via Natural or Artificial Opening Endoscopic	Hysterectomy
0UT9FZZ	ICD-10	Resection of Uterus, Via Natural or Artificial Opening With Percutaneous Endoscopic Assistance	Hysterectomy
0UTC0ZZ	ICD-10	Resection of Cervix, Open Approach	Hysterectomy
0UTC4ZZ	ICD-10	Resection of Cervix, Percutaneous Endoscopic Approach	Hysterectomy
0UTC7ZZ	ICD-10	Resection of Cervix, Via Natural or Artificial Opening	Hysterectomy
0UTC8ZZ	ICD-10	Resection of Cervix, Via Natural or Artificial Opening Endoscopic	Hysterectomy
58150	CPT	Total abdominal hysterectomy (corpus and cervix), with or without removal of tube(s), with or without removal of ovary(s)	Hysterectomy
58180	CPT	Supracervical abdominal hysterectomy (subtotal hysterectomy), with or without removal of tube(s), with or without removal	Hysterectomy
58260	CPT	Vaginal hysterectomy, for uterus 250 g or less	Hysterectomy
58262	CPT	Vaginal hysterectomy, for uterus 250 g or less; with removal of tube(s), and/or ovary(s)	Hysterectomy
58275	CPT	Vaginal hysterectomy, with total or partial vaginectomy	Hysterectomy
58290	CPT	Vaginal hysterectomy, for uterus greater than 250 g	Hysterectomy
58291	CPT	Vaginal hysterectomy, for uterus greater than 250 g; with removal of tube(s) and/or ovary(s)	Hysterectomy
58541	CPT	Laparoscopy, surgical, supracervical hysterectomy, for uterus 250 g or less	Hysterectomy
58542	CPT	Laparoscopy, surgical, supracervical hysterectomy, for uterus 250 g or less; with removal of tube(s) and/or ovary(s)	Hysterectomy
58543	CPT	Laparoscopy, surgical, supracervical hysterectomy, for uterus greater than 250 g	Hysterectomy
58544	CPT	Laparoscopy, surgical, supracervical hysterectomy, for uterus greater than 250 g; with removal of tube(s) and/or ovary(s)	Hysterectomy
58550	CPT	Laparoscopy, surgical, with vaginal hysterectomy, for uterus 250 g or less	Hysterectomy
58552	CPT	Laparoscopy, surgical, with vaginal hysterectomy, for uterus 250 grams or less; with removal of tube(s) and/or ovary(s)	Hysterectomy
58553	CPT	Laparoscopy, surgical, with vaginal hysterectomy, for uterus greater than 250 g	Hysterectomy

58554	CPT	Laparoscopy, surgical, with vaginal hysterectomy, for uterus greater than 250 grams; with removal of tube(s) and/or ovary(s)	Hysterectomy
58570	CPT	Laparoscopy, surgical, with total hysterectomy, for uterus 250 g or less	Hysterectomy
58571	CPT	Laparoscopy, surgical, with total hysterectomy, for uterus 250 g or less; with removal of tube(s) and/or ovary(s)	Hysterectomy
58572	CPT	Laparoscopy, surgical, with total hysterectomy, for uterus greater than 250 g	Hysterectomy
58573	CPT	Laparoscopy, surgical, with total hysterectomy, for uterus greater than 250 g; with removal of tube(s) and/or ovary(s)	Hysterectomy
58661	CPT	Laparoscopy removal of adnexa	Hysterectomy
58720	CPT	Removal of ovary/tube(s)	Hysterectomy
8552	ICD-9	Bilateral injection into breast for augmentation	Mammoplasty
8554	ICD-9	Bilateral breast implant	Mammoplasty
8589	ICD-9	Other mammoplasty	Mammoplasty
0HRT07Z	ICD-10	Replacement of Right Breast with Autologous Tissue Substitute, Open Approach	Mammoplasty
0HRT0KZ	ICD-10	Replacement of Right Breast with Nonautologous Tissue Substitute, Open Approach	Mammoplasty
0HRT37Z	ICD-10	Replacement of Right Breast with Autologous Tissue Substitute, Percutaneous Approach	Mammoplasty
0HRT3KZ	ICD-10	Replacement of Right Breast with Nonautologous Tissue Substitute, Percutaneous Approach	Mammoplasty
0HRTXJZ	ICD-10	Replacement of Right Breast with Synthetic Substitute, External Approach	Mammoplasty
0HRU07Z	ICD-10	Replacement of Left Breast with Autologous Tissue Substitute, Open Approach	Mammoplasty
0HRU0KZ	ICD-10	Replacement of Left Breast with Nonautologous Tissue Substitute, Open Approach	Mammoplasty
0HRU37Z	ICD-10	Replacement of Left Breast with Autologous Tissue Substitute, Percutaneous Approach	Mammoplasty
0HRU3KZ	ICD-10	Replacement of Left Breast with Nonautologous Tissue Substitute, Percutaneous Approach	Mammoplasty
0HRUXJZ	ICD-10	Replacement of Left Breast with Synthetic Substitute, External Approach	Mammoplasty
0HRV07Z	ICD-10	Replacement of Bilateral Breast with Autologous Tissue Substitute, Open Approach	Mammoplasty
0HRV0KZ	ICD-10	Replacement of Bilateral Breast with Nonautologous Tissue Substitute, Open Approach	Mammoplasty
0HRV37Z	ICD-10	Replacement of Bilateral Breast with Autologous Tissue Substitute, Percutaneous Approach	Mammoplasty
0HRV3KZ	ICD-10	Replacement of Bilateral Breast with Nonautologous Tissue Substitute, Percutaneous Approach	Mammoplasty
0HRVXJZ	ICD-10	Replacement of Bilateral Breast with Synthetic Substitute, External Approach	Mammoplasty
0HUT07Z	ICD-10	Supplement Right Breast with Autologous Tissue Substitute, Open Approach	Mammoplasty
0HUT0JZ	ICD-10	Supplement Right Breast with Synthetic Substitute, Open Approach	Mammoplasty
0HUT0KZ	ICD-10	Supplement Right Breast with Nonautologous Tissue Substitute, Open Approach	Mammoplasty
0HUT37Z	ICD-10	Supplement Right Breast with Autologous Tissue Substitute, Percutaneous Approach	Mammoplasty
0HUT3JZ	ICD-10	Supplement Right Breast with Synthetic Substitute, Percutaneous Approach	Mammoplasty
0HUT3KZ	ICD-10	Supplement Right Breast with Nonautologous Tissue Substitute, Percutaneous Approach	Mammoplasty
0HUT77Z	ICD-10	Supplement Right Breast with Autologous Tissue Substitute, Via Natural or Artificial Opening	Mammoplasty
0HUT7JZ	ICD-10	Supplement Right Breast with Synthetic Substitute, Via Natural or Artificial Opening	Mammoplasty
0HUT7KZ	ICD-10	Supplement Right Breast with Nonautologous Tissue Substitute, Via Natural or Artificial Opening	Mammoplasty
0HUT87Z	ICD-10	Supplement Right Breast with Autologous Tissue Substitute, Via Natural or Artificial Opening Endoscopic	Mammoplasty
0HUT8JZ	ICD-10	Supplement Right Breast with Synthetic Substitute, Via Natural or Artificial Opening Endoscopic	Mammoplasty
0HUT8KZ	ICD-10	Supplement Right Breast with Nonautologous Tissue Substitute, Via Natural or Artificial Opening Endoscopic	Mammoplasty
0HUTX7Z	ICD-10	Supplement Right Breast with Autologous Tissue Substitute, External Approach	Mammoplasty
0HUTXJZ	ICD-10	Supplement Right Breast with Synthetic Substitute, External Approach	Mammoplasty
0HUTXKZ	ICD-10	Supplement Right Breast with Nonautologous Tissue Substitute, External Approach	Mammoplasty
0HUU07Z	ICD-10	Supplement Left Breast with Autologous Tissue Substitute, Open Approach	Mammoplasty
0HUU0JZ	ICD-10	Supplement Left Breast with Synthetic Substitute, Open Approach	Mammoplasty
0HUU0KZ	ICD-10	Supplement Left Breast with Nonautologous Tissue Substitute, Open Approach	Mammoplasty
0HUU37Z	ICD-10	Supplement Left Breast with Autologous Tissue Substitute, Percutaneous Approach	Mammoplasty
0HUU3JZ	ICD-10	Supplement Left Breast with Synthetic Substitute, Percutaneous Approach	Mammoplasty
0HUU3KZ	ICD-10	Supplement Left Breast with Nonautologous Tissue Substitute, Percutaneous Approach	Mammoplasty

0HUU77Z	ICD-10	Supplement Left Breast with Autologous Tissue Substitute, Via Natural or Artificial Opening	Mammoplasty
0HUU7JZ	ICD-10	Supplement Left Breast with Synthetic Substitute, Via Natural or Artificial Opening	Mammoplasty
0HUU7KZ	ICD-10	Supplement Left Breast with Nonautologous Tissue Substitute, Via Natural or Artificial Opening	Mammoplasty
0HUU87Z	ICD-10	Supplement Left Breast with Autologous Tissue Substitute, Via Natural or Artificial Opening Endoscopic	Mammoplasty
0HUU8JZ	ICD-10	Supplement Left Breast with Synthetic Substitute, Via Natural or Artificial Opening Endoscopic	Mammoplasty
0HUU8KZ	ICD-10	Supplement Left Breast with Nonautologous Tissue Substitute, Via Natural or Artificial Opening Endoscopic	Mammoplasty
0HUUX7Z	ICD-10	Supplement Left Breast with Autologous Tissue Substitute, External Approach	Mammoplasty
0HUUXJZ	ICD-10	Supplement Left Breast with Synthetic Substitute, External Approach	Mammoplasty
0HUUXKZ	ICD-10	Supplement Left Breast with Nonautologous Tissue Substitute, External Approach	Mammoplasty
0HUV07Z	ICD-10	Supplement Bilateral Breast with Autologous Tissue Substitute, Open Approach	Mammoplasty
0HUV0JZ	ICD-10	Supplement Bilateral Breast with Synthetic Substitute, Open Approach	Mammoplasty
0HUV0KZ	ICD-10	Supplement Bilateral Breast with Nonautologous Tissue Substitute, Open Approach	Mammoplasty
0HUV37Z	ICD-10	Supplement Bilateral Breast with Autologous Tissue Substitute, Percutaneous Approach	Mammoplasty
0HUV3JZ	ICD-10	Supplement Bilateral Breast with Synthetic Substitute, Percutaneous Approach	Mammoplasty
0HUV3KZ	ICD-10	Supplement Bilateral Breast with Nonautologous Tissue Substitute, Percutaneous Approach	Mammoplasty
0HUV77Z	ICD-10	Supplement Bilateral Breast with Autologous Tissue Substitute, Via Natural or Artificial Opening	Mammoplasty
0HUV7JZ	ICD-10	Supplement Bilateral Breast with Synthetic Substitute, Via Natural or Artificial Opening	Mammoplasty
0HUV7KZ	ICD-10	Supplement Bilateral Breast with Nonautologous Tissue Substitute, Via Natural or Artificial Opening	Mammoplasty
0HUV87Z	ICD-10	Supplement Bilateral Breast with Autologous Tissue Substitute, Via Natural or Artificial Opening Endoscopic	Mammoplasty
0HUV8JZ	ICD-10	Supplement Bilateral Breast with Synthetic Substitute, Via Natural or Artificial Opening Endoscopic	Mammoplasty
0HUV8KZ	ICD-10	Supplement Bilateral Breast with Nonautologous Tissue Substitute, Via Natural or Artificial Opening Endoscopic	Mammoplasty
0HUVX7Z	ICD-10	Supplement Bilateral Breast with Autologous Tissue Substitute, External Approach	Mammoplasty
0HUVXJZ	ICD-10	Supplement Bilateral Breast with Synthetic Substitute, External Approach	Mammoplasty
0HUVXKZ	ICD-10	Supplement Bilateral Breast with Nonautologous Tissue Substitute, External Approach	Mammoplasty
19324	CPT	Mammaplasty, augmentation; without prosthetic implant	Mammoplasty
19325	CPT	Mammaplasty, augmentation; with prosthetic implant	Mammoplasty
8523	ICD-9	Subtotal mastectomy	Mastectomy
8532	ICD-9	Bilateral reduction mammoplasty	Mastectomy
8536	ICD-9	Other bilateral subcutaneous mammectomy	Mastectomy
8542	ICD-9	Bilateral simple mastectomy	Mastectomy
8544	ICD-9	Bilateral extended simple mastectomy	Mastectomy
8546	ICD-9	Bilateral radical mastectomy	Mastectomy
8548	ICD-9	Bilateral extended radical mastectomy	Mastectomy
19301	CPT	Mastectomy, partial (eg, lumpectomy, tylectomy, quadrantectomy, segmentectomy)	Mastectomy
19303	CPT	Mastectomy, simple, complete	Mastectomy
19304	CPT	Mastectomy, subcutaneous	Mastectomy
19305	CPT	Mastectomy, radical	Mastectomy
19318	CPT	Reduction of large breast	Mastectomy
6241	ICD-9	Removal of both testes at same operative episode	Orchiectomy
6242	ICD-9	Removal of remaining testis	Orchiectomy
0VTC0ZZ	ICD-10	Resection of Bilateral Testes, Open Approach	Orchiectomy
0VTC4ZZ	ICD-10	Resection of Bilateral Testes, Percutaneous Endoscopic Approach	Orchiectomy
54520	CPT	Orchiectomy, simple (including subcapsular), with or without testicular prosthesis, scrotal or inguinal approach	Orchiectomy
54522	CPT	Orchiectomy partial	Orchiectomy
54530	CPT	Removal of testis	Orchiectomy
54690	CPT	Laparoscopy, surgical; orchiectomy	Orchiectomy

627	ICD-9	Insertion of testicular prosthesis	Phalloplasty
704	ICD-9	Obliteration and total excision of vagina	Phalloplasty
708	ICD-9	Obliteration of vaginal vault	Phalloplasty
6443	ICD-9	Construction of penis	Phalloplasty
0VHD0YZ	ICD-10	Insertion of Other Device into Testis, Open Approach	Phalloplasty
0VRC0JZ	ICD-10	Replacement of Bilateral Testes with Synthetic Substitute, Open Approach	Phalloplasty
0W4N071	ICD-10	Creation of Penis in Female Perineum with Autologous Tissue Substitute, Open Approach	Phalloplasty
0W4N0J1	ICD-10	Creation of Penis in Female Perineum with Synthetic Substitute, Open Approach	Phalloplasty
0W4N0K1	ICD-10	Creation of Penis in Female Perineum with Nonautologous Tissue Substitute, Open Approach	Phalloplasty
0W4N0Z1	ICD-10	Creation of Penis in Female Perineum, Open Approach	Phalloplasty
54400	CPT	Insert semi-rigid prosthesis	Phalloplasty
54401	CPT	Insert self-contained prosthesis	Phalloplasty
54405	CPT	Insert multi-component penis prosthesis	Phalloplasty
54406	CPT	Remove multi-component penis prosthesis	Phalloplasty
54410	CPT	Remove/replace multi-component penis prosthesis	Phalloplasty
54415	CPT	Remove self-contained penis prosthesis	Phalloplasty
54416	CPT	Remove/replace self-contained penis prosthesis	Phalloplasty
55980	CPT	Sex transformation female to male	Phalloplasty
643	ICD-9	Amputation of penis	Vaginoplasty
7061	ICD-9	Vaginal construction	Vaginoplasty
7063	ICD-9	Vaginal construction with graft or prosthesis	Vaginoplasty
0VTS0ZZ	ICD-10	Resection of Penis, Open Approach	Vaginoplasty
0VTS4ZZ	ICD-10	Resection of Penis, Percutaneous Endoscopic Approach	Vaginoplasty
0W4M070	ICD-10	Creation of Vagina in Male Perineum with Autologous Tissue Substitute, Open Approach	Vaginoplasty
0W4M0J0	ICD-10	Creation of Vagina in Male Perineum with Synthetic Substitute, Open Approach	Vaginoplasty
0W4M0K0	ICD-10	Creation of Vagina in Male Perineum with Nonautologous Tissue Substitute, Open Approach	Vaginoplasty
0W4M0Z0	ICD-10	Creation of Vagina in Male Perineum, Open Approach	Vaginoplasty
54120	CPT	Amputation of penis; partial	Vaginoplasty
54125	CPT	Amputation of penis; complete	Vaginoplasty
55150	CPT	Removal of scrotum	Vaginoplasty
55970	CPT	Sex transformation, male to female	Vaginoplasty
57291	CPT	Construction of artificial vagina; without graft	Vaginoplasty
57292	CPT	Construction of artificial vagina; with graft	Vaginoplasty
57295	CPT	Revise vaginal graft via vagina	Vaginoplasty
8587	ICD-9	Other repair or reconstruction of nipple	Unspecified Chest
0H0T0ZZ	ICD-10	Alteration of Right Breast, Open Approach	Unspecified Chest
0H0T3ZZ	ICD-10	Alteration of Right Breast, Percutaneous Approach	Unspecified Chest
0H0TXZZ	ICD-10	Alteration of Right Breast, External Approach	Unspecified Chest
0H0U0ZZ	ICD-10	Alteration of Left Breast, Open Approach	Unspecified Chest
0H0U3ZZ	ICD-10	Alteration of Left Breast, Percutaneous Approach	Unspecified Chest
0H0UXZZ	ICD-10	Alteration of Left Breast, External Approach	Unspecified Chest
0H0V07Z	ICD-10	Alteration of Bilateral Breast with Autologous Tissue Substitute, Open Approach	Unspecified Chest
0H0V0JZ	ICD-10	Alteration of Bilateral Breast with Synthetic Substitute, Open Approach	Unspecified Chest
0H0V0KZ	ICD-10	Alteration of Bilateral Breast with Nonautologous Tissue Substitute, Open Approach	Unspecified Chest
0H0V0ZZ	ICD-10	Alteration of Bilateral Breast, Open Approach	Unspecified Chest
0H0V37Z	ICD-10	Alteration of Bilateral Breast with Autologous Tissue Substitute, Percutaneous Approach	Unspecified Chest

0H0V3JZ	ICD-10	Alteration of Bilateral Breast with Synthetic Substitute, Percutaneous Approach	Unspecified Chest
0H0V3KZ	ICD-10	Alteration of Bilateral Breast with Nonautologous Tissue Substitute, Percutaneous Approach	Unspecified Chest
0H0V3ZZ	ICD-10	Alteration of Bilateral Breast, Percutaneous Approach	Unspecified Chest
0H0VXZZ	ICD-10	Alteration of Bilateral Breast, External Approach	Unspecified Chest
0HBV0ZZ	ICD-10	Excision of Bilateral Breast, Open Approach	Unspecified Chest
0HBW0ZZ	ICD-10	Excision of Right Nipple, Open Approach	Unspecified Chest
0HBX0ZZ	ICD-10	Excision of Left Nipple, Open Approach	Unspecified Chest
0HMTXZZ	ICD-10	Reattachment of Right Breast, External Approach	Unspecified Chest
0HMUXZZ	ICD-10	Reattachment of Left Breast, External Approach	Unspecified Chest
0HMVXZZ	ICD-10	Reattachment of Bilateral Breast, External Approach	Unspecified Chest
0HMWXZZ	ICD-10	Reattachment of Right Nipple, External Approach	Unspecified Chest
0HMXXZZ	ICD-10	Reattachment of Left Nipple, External Approach	Unspecified Chest
0HNT0ZZ	ICD-10	Release Right Breast, Open Approach	Unspecified Chest
0HNT3ZZ	ICD-10	Release Right Breast, Percutaneous Approach	Unspecified Chest
0HNT7ZZ	ICD-10	Release Right Breast, Via Natural or Artificial Opening	Unspecified Chest
0HNT8ZZ	ICD-10	Release Right Breast, Via Natural or Artificial Opening Endoscopic	Unspecified Chest
0HNTXZZ	ICD-10	Release Right Breast, External Approach	Unspecified Chest
0HNU0ZZ	ICD-10	Release Left Breast, Open Approach	Unspecified Chest
0HNU3ZZ	ICD-10	Release Left Breast, Percutaneous Approach	Unspecified Chest
0HNU7ZZ	ICD-10	Release Left Breast, Via Natural or Artificial Opening	Unspecified Chest
0HNU8ZZ	ICD-10	Release Left Breast, Via Natural or Artificial Opening Endoscopic	Unspecified Chest
0HNUXZZ	ICD-10	Release Left Breast, External Approach	Unspecified Chest
0HNV0ZZ	ICD-10	Release Bilateral Breast, Open Approach	Unspecified Chest
0HNV3ZZ	ICD-10	Release Bilateral Breast, Percutaneous Approach	Unspecified Chest
0HNV7ZZ	ICD-10	Release Bilateral Breast, Via Natural or Artificial Opening	Unspecified Chest
0HNV8ZZ	ICD-10	Release Bilateral Breast, Via Natural or Artificial Opening Endoscopic	Unspecified Chest
0HNVXZZ	ICD-10	Release Bilateral Breast, External Approach	Unspecified Chest
0HNW0ZZ	ICD-10	Release Right Nipple, Open Approach	Unspecified Chest
0HNW3ZZ	ICD-10	Release Right Nipple, Percutaneous Approach	Unspecified Chest
0HNW7ZZ	ICD-10	Release Right Nipple, Via Natural or Artificial Opening	Unspecified Chest
0HNW8ZZ	ICD-10	Release Right Nipple, Via Natural or Artificial Opening Endoscopic	Unspecified Chest
0HNWXZZ	ICD-10	Release Right Nipple, External Approach	Unspecified Chest
0HNX0ZZ	ICD-10	Release Left Nipple, Open Approach	Unspecified Chest
0HNX3ZZ	ICD-10	Release Left Nipple, Percutaneous Approach	Unspecified Chest
0HNX7ZZ	ICD-10	Release Left Nipple, Via Natural or Artificial Opening	Unspecified Chest
0HNX8ZZ	ICD-10	Release Left Nipple, Via Natural or Artificial Opening Endoscopic	Unspecified Chest
0HNXXZZ	ICD-10	Release Left Nipple, External Approach	Unspecified Chest
0HQT0ZZ	ICD-10	Repair Right Breast, Open Approach	Unspecified Chest
0HQT3ZZ	ICD-10	Repair Right Breast, Percutaneous Approach	Unspecified Chest
0HQT7ZZ	ICD-10	Repair Right Breast, Via Natural or Artificial Opening	Unspecified Chest
0HQT8ZZ	ICD-10	Repair Right Breast, Via Natural or Artificial Opening Endoscopic	Unspecified Chest
0HQTXZZ	ICD-10	Repair Right Breast, External Approach	Unspecified Chest
0HQU0ZZ	ICD-10	Repair Left Breast, Open Approach	Unspecified Chest
0HQU3ZZ	ICD-10	Repair Left Breast, Percutaneous Approach	Unspecified Chest
0HQU7ZZ	ICD-10	Repair Left Breast, Via Natural or Artificial Opening	Unspecified Chest
0HQU8ZZ	ICD-10	Repair Left Breast, Via Natural or Artificial Opening Endoscopic	Unspecified Chest

0HQUXZZ	ICD-10	Repair Left Breast, External Approach	Unspecified Chest
0HQV0ZZ	ICD-10	Repair Bilateral Breast, Open Approach	Unspecified Chest
0HQV3ZZ	ICD-10	Repair Bilateral Breast, Percutaneous Approach	Unspecified Chest
0HQV7ZZ	ICD-10	Repair Bilateral Breast, Via Natural or Artificial Opening	Unspecified Chest
0HQV8ZZ	ICD-10	Repair Bilateral Breast, Via Natural or Artificial Opening Endoscopic	Unspecified Chest
0HQVXZZ	ICD-10	Repair Bilateral Breast, External Approach	Unspecified Chest
0HQW0ZZ	ICD-10	Repair Right Nipple, Open Approach	Unspecified Chest
0HQW3ZZ	ICD-10	Repair Right Nipple, Percutaneous Approach	Unspecified Chest
0HQW7ZZ	ICD-10	Repair Right Nipple, Via Natural or Artificial Opening	Unspecified Chest
0HQW8ZZ	ICD-10	Repair Right Nipple, Via Natural or Artificial Opening Endoscopic	Unspecified Chest
0HQWXZZ	ICD-10	Repair Right Nipple, External Approach	Unspecified Chest
0HQX0ZZ	ICD-10	Repair Left Nipple, Open Approach	Unspecified Chest
0HQX3ZZ	ICD-10	Repair Left Nipple, Percutaneous Approach	Unspecified Chest
0HQX7ZZ	ICD-10	Repair Left Nipple, Via Natural or Artificial Opening	Unspecified Chest
0HQX8ZZ	ICD-10	Repair Left Nipple, Via Natural or Artificial Opening Endoscopic	Unspecified Chest
0HQXXZZ	ICD-10	Repair Left Nipple, External Approach	Unspecified Chest
0HQY0ZZ	ICD-10	Repair Supernumerary Breast, Open Approach	Unspecified Chest
0HQY3ZZ	ICD-10	Repair Supernumerary Breast, Percutaneous Approach	Unspecified Chest
0HQY7ZZ	ICD-10	Repair Supernumerary Breast, Via Natural or Artificial Opening	Unspecified Chest
0HQY8ZZ	ICD-10	Repair Supernumerary Breast, Via Natural or Artificial Opening Endoscopic	Unspecified Chest
0HQYXZZ	ICD-10	Repair Supernumerary Breast, External Approach	Unspecified Chest
0HRW07Z	ICD-10	Replacement of Right Nipple with Autologous Tissue Substitute, Open Approach	Unspecified Chest
0HRW0JZ	ICD-10	Replacement of Right Nipple with Synthetic Substitute, Open Approach	Unspecified Chest
0HRW0KZ	ICD-10	Replacement of Right Nipple with Nonautologous Tissue Substitute, Open Approach	Unspecified Chest
0HRW37Z	ICD-10	Replacement of Right Nipple with Autologous Tissue Substitute, Percutaneous Approach	Unspecified Chest
0HRW3JZ	ICD-10	Replacement of Right Nipple with Synthetic Substitute, Percutaneous Approach	Unspecified Chest
0HRW3KZ	ICD-10	Replacement of Right Nipple with Nonautologous Tissue Substitute, Percutaneous Approach	Unspecified Chest
0HRWX7Z	ICD-10	Replacement of Right Nipple with Autologous Tissue Substitute, External Approach	Unspecified Chest
0HRWXJZ	ICD-10	Replacement of Right Nipple with Synthetic Substitute, External Approach	Unspecified Chest
0HRWXKZ	ICD-10	Replacement of Right Nipple with Nonautologous Tissue Substitute, External Approach	Unspecified Chest
0HRX07Z	ICD-10	Replacement of Left Nipple with Autologous Tissue Substitute, Open Approach	Unspecified Chest
0HRX0JZ	ICD-10	Replacement of Left Nipple with Synthetic Substitute, Open Approach	Unspecified Chest
0HRX0KZ	ICD-10	Replacement of Left Nipple with Nonautologous Tissue Substitute, Open Approach	Unspecified Chest
0HRX37Z	ICD-10	Replacement of Left Nipple with Autologous Tissue Substitute, Percutaneous Approach	Unspecified Chest
0HRX3JZ	ICD-10	Replacement of Left Nipple with Synthetic Substitute, Percutaneous Approach	Unspecified Chest
0HRX3KZ	ICD-10	Replacement of Left Nipple with Nonautologous Tissue Substitute, Percutaneous Approach	Unspecified Chest
0HRXX7Z	ICD-10	Replacement of Left Nipple with Autologous Tissue Substitute, External Approach	Unspecified Chest
0HRXXJZ	ICD-10	Replacement of Left Nipple with Synthetic Substitute, External Approach	Unspecified Chest
0HRXXKZ	ICD-10	Replacement of Left Nipple with Nonautologous Tissue Substitute, External Approach	Unspecified Chest
0HSV0ZZ	ICD-10	Reposition Bilateral Breast, Open Approach	Unspecified Chest
0HSWXZZ	ICD-10	Reposition Right Nipple, External Approach	Unspecified Chest
0HSXXZZ	ICD-10	Reposition Left Nipple, External Approach	Unspecified Chest
0HTV0ZZ	ICD-10	Resection of Bilateral Breast, Open Approach	Unspecified Chest
0HUW07Z	ICD-10	Supplement Right Nipple with Autologous Tissue Substitute, Open Approach	Unspecified Chest
0HUW0JZ	ICD-10	Supplement Right Nipple with Synthetic Substitute, Open Approach	Unspecified Chest
0HUW0KZ	ICD-10	Supplement Right Nipple with Nonautologous Tissue Substitute, Open Approach	Unspecified Chest

0HUW37Z	ICD-10	Supplement Right Nipple with Autologous Tissue Substitute, Percutaneous Approach	Unspecified Chest
0HUW3JZ	ICD-10	Supplement Right Nipple with Synthetic Substitute, Percutaneous Approach	Unspecified Chest
0HUW3KZ	ICD-10	Supplement Right Nipple with Nonautologous Tissue Substitute, Percutaneous Approach	Unspecified Chest
0HUW77Z	ICD-10	Supplement Right Nipple with Autologous Tissue Substitute, Via Natural or Artificial Opening	Unspecified Chest
0HUW7JZ	ICD-10	Supplement Right Nipple with Synthetic Substitute, Via Natural or Artificial Opening	Unspecified Chest
0HUW7KZ	ICD-10	Supplement Right Nipple with Nonautologous Tissue Substitute, Via Natural or Artificial Opening	Unspecified Chest
0HUW87Z	ICD-10	Supplement Right Nipple with Autologous Tissue Substitute, Via Natural or Artificial Opening Endoscopic	Unspecified Chest
0HUW8JZ	ICD-10	Supplement Right Nipple with Synthetic Substitute, Via Natural or Artificial Opening Endoscopic	Unspecified Chest
0HUW8KZ	ICD-10	Supplement Right Nipple with Nonautologous Tissue Substitute, Via Natural or Artificial Opening Endoscopic	Unspecified Chest
0HUWX7Z	ICD-10	Supplement Right Nipple with Autologous Tissue Substitute, External Approach	Unspecified Chest
0HUWXJZ	ICD-10	Supplement Right Nipple with Synthetic Substitute, External Approach	Unspecified Chest
0HUWXKZ	ICD-10	Supplement Right Nipple with Nonautologous Tissue Substitute, External Approach	Unspecified Chest
0HUX07Z	ICD-10	Supplement Left Nipple with Autologous Tissue Substitute, Open Approach	Unspecified Chest
0HUX0JZ	ICD-10	Supplement Left Nipple with Synthetic Substitute, Open Approach	Unspecified Chest
0HUX0KZ	ICD-10	Supplement Left Nipple with Nonautologous Tissue Substitute, Open Approach	Unspecified Chest
0HUX37Z	ICD-10	Supplement Left Nipple with Autologous Tissue Substitute, Percutaneous Approach	Unspecified Chest
0HUX3JZ	ICD-10	Supplement Left Nipple with Synthetic Substitute, Percutaneous Approach	Unspecified Chest
0HUX3KZ	ICD-10	Supplement Left Nipple with Nonautologous Tissue Substitute, Percutaneous Approach	Unspecified Chest
0HUX77Z	ICD-10	Supplement Left Nipple with Autologous Tissue Substitute, Via Natural or Artificial Opening	Unspecified Chest
0HUX7JZ	ICD-10	Supplement Left Nipple with Synthetic Substitute, Via Natural or Artificial Opening	Unspecified Chest
0HUX7KZ	ICD-10	Supplement Left Nipple with Nonautologous Tissue Substitute, Via Natural or Artificial Opening	Unspecified Chest
0HUX87Z	ICD-10	Supplement Left Nipple with Autologous Tissue Substitute, Via Natural or Artificial Opening Endoscopic	Unspecified Chest
0HUX8JZ	ICD-10	Supplement Left Nipple with Synthetic Substitute, Via Natural or Artificial Opening Endoscopic	Unspecified Chest
0HUX8KZ	ICD-10	Supplement Left Nipple with Nonautologous Tissue Substitute, Via Natural or Artificial Opening Endoscopic	Unspecified Chest
0HUXX7Z	ICD-10	Supplement Left Nipple with Autologous Tissue Substitute, External Approach	Unspecified Chest
0HUXXJZ	ICD-10	Supplement Left Nipple with Synthetic Substitute, External Approach	Unspecified Chest
0HUXXKZ	ICD-10	Supplement Left Nipple with Nonautologous Tissue Substitute, External Approach	Unspecified Chest
19300	CPT	Removal of breast tissue	Unspecified Chest
19316	CPT	Mastopexy	Unspecified Chest
19340	CPT	Immediate breast prosthesis	Unspecified Chest
19342	CPT	Delayed breast prosthesis	Unspecified Chest
19350	CPT	Nipple/areola reconstruction	Unspecified Chest
19357	CPT	Breast reconstruction, immediate or delayed, with tissue expander, including subsequent expansion	Unspecified Chest
19364	CPT	Breast reconstruction	Unspecified Chest
19366	CPT	Breast reconstruction	Unspecified Chest
19370	CPT	Surgery of breast capsule	Unspecified Chest
19380	CPT	Breast reconstruction, immediate or delayed, with tissue expander, including subsequent expansion	Unspecified Chest
19499	CPT	Breast surgery procedure	Unspecified Chest
645	ICD-9	Operations for sex transformation, not elsewhere classified	Unspecified Genital
714	ICD-9	Operations on clitoris	Unspecified Genital
6149	ICD-9	Other repair of scrotum and tunica vaginalis	Unspecified Genital
6449	ICD-9	Other repair of penis	Unspecified Genital
7162	ICD-9	Bilateral vulvectomy	Unspecified Genital
0U5J0ZZ	ICD-10	Destruction of Clitoris, Open Approach	Unspecified Genital
0U5JXZZ	ICD-10	Destruction of Clitoris, External Approach	Unspecified Genital
0U9J00Z	ICD-10	Drainage of Clitoris with Drainage Device, Open Approach	Unspecified Genital

0U9J0ZZ	ICD-10	Drainage of Clitoris, Open Approach	Unspecified Genital
0U9JX0Z	ICD-10	Drainage of Clitoris with Drainage Device, External Approach	Unspecified Genital
0U9JXZZ	ICD-10	Drainage of Clitoris, External Approach	Unspecified Genital
0UBG0ZZ	ICD-10	Excision of Vagina, Open Approach	Unspecified Genital
0UBG4ZZ	ICD-10	Excision of Vagina, Percutaneous Endoscopic Approach	Unspecified Genital
0UBJ0ZZ	ICD-10	Excision of Clitoris, Open Approach	Unspecified Genital
0UBJXZZ	ICD-10	Excision of Clitoris, External Approach	Unspecified Genital
0UBM0ZZ	ICD-10	Excision of Vulva, Open Approach	Unspecified Genital
0UBMXZZ	ICD-10	Excision of Vulva, External Approach	Unspecified Genital
0UCJ0ZZ	ICD-10	Extirpation of Matter from Clitoris, Open Approach	Unspecified Genital
0UCJXZZ	ICD-10	Extirpation of Matter from Clitoris, External Approach	Unspecified Genital
0ULG7ZZ	ICD-10	Occlusion of Vagina, Via Natural or Artificial Opening	Unspecified Genital
0UMJXZZ	ICD-10	Reattachment of Clitoris, External Approach	Unspecified Genital
0UNG7ZZ	ICD-10	Release Vagina, Via Natural or Artificial Opening	Unspecified Genital
0UNJ0ZZ	ICD-10	Release Clitoris, Open Approach	Unspecified Genital
0UNJXZZ	ICD-10	Release Clitoris, External Approach	Unspecified Genital
0UQG0ZZ	ICD-10	Repair Vagina, Open Approach	Unspecified Genital
0UQG7ZZ	ICD-10	Repair Vagina, Via Natural or Artificial Opening	Unspecified Genital
0UQGXZZ	ICD-10	Repair Vagina, External Approach	Unspecified Genital
0UQJ0ZZ	ICD-10	Repair Clitoris, Open Approach	Unspecified Genital
0UQJXZZ	ICD-10	Repair Clitoris, External Approach	Unspecified Genital
0UQMXZZ	ICD-10	Repair Vulva, External Approach	Unspecified Genital
0USG4ZZ	ICD-10	Reposition Vagina, Percutaneous Endoscopic Approach	Unspecified Genital
0UTG0ZZ	ICD-10	Resection of Vagina, Open Approach	Unspecified Genital
0UTG4ZZ	ICD-10	Resection of Vagina, Percutaneous Endoscopic Approach	Unspecified Genital
0UTG7ZZ	ICD-10	Resection of Vagina, Via Natural or Artificial Opening	Unspecified Genital
0UTG8ZZ	ICD-10	Resection of Vagina, Via Natural or Artificial Opening Endoscopic	Unspecified Genital
0UTJ0ZZ	ICD-10	Resection of Clitoris, Open Approach	Unspecified Genital
0UTJXZZ	ICD-10	Resection of Clitoris, External Approach	Unspecified Genital
0UTM0ZZ	ICD-10	Resection of Vulva, Open Approach	Unspecified Genital
0UTMXZZ	ICD-10	Resection of Vulva, External Approach	Unspecified Genital
0UUG07Z	ICD-10	Supplement Vagina with Autologous Tissue Substitute, Open Approach	Unspecified Genital
0UUG0JZ	ICD-10	Supplement Vagina with Synthetic Substitute, Open Approach	Unspecified Genital
0UUG0KZ	ICD-10	Supplement Vagina with Nonautologous Tissue Substitute, Open Approach	Unspecified Genital
0UUG47Z	ICD-10	Supplement Vagina with Autologous Tissue Substitute, Percutaneous Endoscopic Approach	Unspecified Genital
0UUG4JZ	ICD-10	Supplement Vagina with Synthetic Substitute, Percutaneous Endoscopic Approach	Unspecified Genital
0UUG4KZ	ICD-10	Supplement Vagina with Nonautologous Tissue Substitute, Percutaneous Endoscopic Approach	Unspecified Genital
0UUG77Z	ICD-10	Supplement Vagina with Autologous Tissue Substitute, Via Natural or Artificial Opening	Unspecified Genital
0UUG7JZ	ICD-10	Supplement Vagina with Synthetic Substitute, Via Natural or Artificial Opening	Unspecified Genital
0UUG7KZ	ICD-10	Supplement Vagina with Nonautologous Tissue Substitute, Via Natural or Artificial Opening	Unspecified Genital
0UUG87Z	ICD-10	Supplement Vagina with Autologous Tissue Substitute, Via Natural or Artificial Opening Endoscopic	Unspecified Genital
0UUG8JZ	ICD-10	Supplement Vagina with Synthetic Substitute, Via Natural or Artificial Opening Endoscopic	Unspecified Genital
0UUG8KZ	ICD-10	Supplement Vagina with Nonautologous Tissue Substitute, Via Natural or Artificial Opening Endoscopic	Unspecified Genital
0UUGX7Z	ICD-10	Supplement Vagina with Autologous Tissue Substitute, External Approach	Unspecified Genital
0UUGXJZ	ICD-10	Supplement Vagina with Synthetic Substitute, External Approach	Unspecified Genital
0UUGXKZ	ICD-10	Supplement Vagina with Nonautologous Tissue Substitute, External Approach	Unspecified Genital

0UUJ07Z	ICD-10	Supplement Clitoris with Autologous Tissue Substitute, Open Approach	Unspecified Genital
0UUJ0JZ	ICD-10	Supplement Clitoris with Synthetic Substitute, Open Approach	Unspecified Genital
0UUJ0KZ	ICD-10	Supplement Clitoris with Nonautologous Tissue Substitute, Open Approach	Unspecified Genital
0UUJX7Z	ICD-10	Supplement Clitoris with Autologous Tissue Substitute, External Approach	Unspecified Genital
0UUJXJZ	ICD-10	Supplement Clitoris with Synthetic Substitute, External Approach	Unspecified Genital
0UUJXKZ	ICD-10	Supplement Clitoris with Nonautologous Tissue Substitute, External Approach	Unspecified Genital
0UUM07Z	ICD-10	Supplement Vulva with Autologous Tissue Substitute, Open Approach	Unspecified Genital
0UWH47Z	ICD-10	Revision of Autologous Tissue Substitute in Vagina and Cul-de-sac, Percutaneous Endoscopic Approach	Unspecified Genital
0UWH77Z	ICD-10	Revision of Autologous Tissue Substitute in Vagina and Cul-de-sac, Via Natural or Artificial Opening	Unspecified Genital
0VB50ZZ	ICD-10	Excision of Scrotum, Open Approach	Unspecified Genital
0VB5XZZ	ICD-10	Excision of Scrotum, External Approach	Unspecified Genital
0VBC0ZZ	ICD-10	Excision of Bilateral Testes, Open Approach	Unspecified Genital
0VBS0ZZ	ICD-10	Excision of Penis, Open Approach	Unspecified Genital
0VPS0JZ	ICD-10	Removal of Synthetic Substitute from Penis, Open Approach	Unspecified Genital
0VQ50ZZ	ICD-10	Repair Scrotum, Open Approach	Unspecified Genital
0VQ5XZZ	ICD-10	Repair Scrotum, External Approach	Unspecified Genital
0VT50ZZ	ICD-10	Resection of Scrotum, Open Approach	Unspecified Genital
0VT90ZZ	ICD-10	Resection of Right Testis, Open Approach	Unspecified Genital
0VT94ZZ	ICD-10	Resection of Right Testis, Percutaneous Endoscopic Approach	Unspecified Genital
0VTB0ZZ	ICD-10	Resection of Left Testis, Open Approach	Unspecified Genital
0VTB4ZZ	ICD-10	Resection of Left Testis, Percutaneous Endoscopic Approach	Unspecified Genital
0VTSXZZ	ICD-10	Resection of Penis, External Approach	Unspecified Genital
0VU5X7Z	ICD-10	Supplement Scrotum with Autologous Tissue Substitute, External Approach	Unspecified Genital
0VUC0JZ	ICD-10	Supplement Bilateral Testes with Synthetic Substitute, Open Approach	Unspecified Genital
0VUS07Z	ICD-10	Supplement Penis with Autologous Tissue Substitute, Open Approach	Unspecified Genital
0VUS0JZ	ICD-10	Supplement Penis with Synthetic Substitute, Open Approach	Unspecified Genital
0VUS0KZ	ICD-10	Supplement Penis with Nonautologous Tissue Substitute, Open Approach	Unspecified Genital
0VUS47Z	ICD-10	Supplement Penis with Autologous Tissue Substitute, Percutaneous Endoscopic Approach	Unspecified Genital
0VUS4JZ	ICD-10	Supplement Penis with Synthetic Substitute, Percutaneous Endoscopic Approach	Unspecified Genital
0VUS4KZ	ICD-10	Supplement Penis with Nonautologous Tissue Substitute, Percutaneous Endoscopic Approach	Unspecified Genital
0VUSX7Z	ICD-10	Supplement Penis with Autologous Tissue Substitute, External Approach	Unspecified Genital
0VUSXJZ	ICD-10	Supplement Penis with Synthetic Substitute, External Approach	Unspecified Genital
0VUSXKZ	ICD-10	Supplement Penis with Nonautologous Tissue Substitute, External Approach	Unspecified Genital
0WPN0JZ	ICD-10	Removal of Synthetic Substitute from Female Perineum, Open Approach	Unspecified Genital
0WUN07Z	ICD-10	Supplement Female Perineum with Autologous Tissue Substitute, Open Approach	Unspecified Genital
50715	CPT	Release of ureter	Unspecified Genital
53010	CPT	Incision of urethra	Unspecified Genital
53020	CPT	Incision of urethra	Unspecified Genital
53210	CPT	Removal of urethra	Unspecified Genital
53240	CPT	Surgery for urethra pouch	Unspecified Genital
53400	CPT	Revise urethra stage 1	Unspecified Genital
53405	CPT	Revise urethra stage 2	Unspecified Genital
53410	CPT	Reconstruction of urethra	Unspecified Genital
53415	CPT	Reconstruction of urethra	Unspecified Genital
53420	CPT	Reconstruct urethra stage 1	Unspecified Genital
53425	CPT	Reconstruct urethra stage 2	Unspecified Genital

53430	CPT	Urethroplasty, reconstruction of female urethra	Unspecified Genital
53460	CPT	Revision of urethra	Unspecified Genital
53520	CPT	Repair of urethra defect	Unspecified Genital
53899	CPT	Urology surgery procedure	Unspecified Genital
54300	CPT	Revision of penis	Unspecified Genital
54304	CPT	Revision of penis	Unspecified Genital
54352	CPT	Reconstruct urethra/penis	Unspecified Genital
54360	CPT	Penis plastic surgery	Unspecified Genital
54660	CPT	Revision of testis	Unspecified Genital
55175	CPT	Scrotoplasty; simple	Unspecified Genital
55180	CPT	Scrotoplasty; complex	Unspecified Genital
55899	CPT	Genital surgery procedure	Unspecified Genital
56620	CPT	Vulvectomy; simple	Unspecified Genital
56625	CPT	Vulvectomy; complete	Unspecified Genital
56800	CPT	Plastic repair of introitus	Unspecified Genital
56805	CPT	Clitoroplasty for intersex state	Unspecified Genital
56810	CPT	Perineoplasty, repair of perineum, nonobstetrical	Unspecified Genital
57106	CPT	Vaginectomy; partial removal of vaginal wall	Unspecified Genital
57107	CPT	Vaginectomy; with removal of paravaginal tissue (radical vaginectomy)	Unspecified Genital
57110	CPT	Vaginectomy; complete removal of vaginal wall	Unspecified Genital
57111	CPT	Vaginectomy; with removal of paravaginal tissue (radical vaginectomy)	Unspecified Genital
57120	CPT	Closure of vagina	Unspecified Genital
57200	CPT	Repair of vagina	Unspecified Genital
57210	CPT	Repair vagina/perineum	Unspecified Genital
57335	CPT	Repair vagina	Unspecified Genital
58999	CPT	Genital surgery procedure	Unspecified Genital
